# Isotypes of autoantibodies against novel differential 4-hydroxy-2-nonenal-modified peptide adducts in serum is associated with rheumatoid arthritis in Taiwanese women

**DOI:** 10.1186/s12911-020-01380-y

**Published:** 2021-02-10

**Authors:** Kai-Leun Tsai, Che-Chang Chang, Yu-Sheng Chang, Yi-Ying Lu, I-Jung Tsai, Jin-Hua Chen, Sheng-Hong Lin, Chih-Chun Tai, Yi-Fang Lin, Hui-Wen Chang, Ching-Yu Lin, Emily Chia-Yu Su

**Affiliations:** 1 Division of Allergy, Immunology and Rheumatology, Department of Internal Medicine, Shuang Ho Hospital, Taipei Medical University, New Taipei City, 23561 Taiwan; 2 Division of Allergy, Immunology and Rheumatology, Department of Internal Medicine, School of Medicine, College of Medicine, Taipei Medical University, Taipei, 11031 Taiwan; 3 Graduate Institute of Translational Medicine, College of Medical Science and Technology, Taipei Medical University, Taipei, 11031 Taiwan; 4 School of Medical Laboratory Science and Biotechnology, College of Medical Science and Technology, Taipei Medical University, 250 Wuxing Street, Taipei, 11031 Taiwan; 5 Graduate Institute of Data Science, College of Management, Taipei Medical University, Taipei, 11031 Taiwan; 6 Research Center of Biostatistics, College of Management, Taipei Medical University, Taipei, 11031 Taiwan; 7 Department of Laboratory Medicine, Taipei Medical University-Shuang-Ho Hospital, Taipei Medical University, New Taipei City, 23561 Taiwan; 8 Department of Medical Laboratory, Taipei Medical University Hospital, Taipei, 11031 Taiwan; 9 PhD Program in Medical Biotechnology, College of Medical Science and Technology, Taipei Medical University, Taipei, 11031 Taiwan; 10 Department of Biotechnology and Animal Science, National Ilan University, Ilan, 26047 Taiwan; 11 Graduate Institute of Biomedical Informatics, College of Medical Science and Technology, Taipei Medical University, Taipei, 11031 Taiwan; 12 Clinical Big Data Research Center, Taipei Medical University Hospital, Taipei, 11031 Taiwan

**Keywords:** Rheumatoid arthritis, 4-hydroxy-2-nonenal, Autoantibody isotype, Serum

## Abstract

**Background:**

Rheumatoid arthritis (RA) is an autoimmune disorder with systemic inflammation and may be induced by oxidative stress that affects an inflamed joint. Our objectives were to examine isotypes of autoantibodies against 4-hydroxy-2-nonenal (HNE) modifications in RA and associate them with increased levels of autoantibodies in RA patients.

**Methods:**

Serum samples from 155 female patients [60 with RA, 35 with osteoarthritis (OA), and 60 healthy controls (HCs)] were obtained. Four novel differential HNE-modified peptide adducts, complement factor H (CFAH)^1211–1230^, haptoglobin (HPT)^78–108^, immunoglobulin (Ig) kappa chain C region (IGKC)^2–19^, and prothrombin (THRB)^328–345^, were re-analyzed using tandem mass spectrometric (MS/MS) spectra (ProteomeXchange: PXD004546) from RA patients vs*.* HCs. Further, we determined serum protein levels of CFAH, HPT, IGKC and THRB, HNE-protein adducts, and autoantibodies against unmodified and HNE-modified peptides. Significant correlations and odds ratios (ORs) were calculated.

**Results:**

Levels of HPT in RA patients were greatly higher than the levels in HCs. Levels of HNE-protein adducts and autoantibodies in RA patients were significantly greater than those of HCs. IgM anti-HPT^78−108^ HNE, IgM anti-IGKC^2−19^, and IgM anti-IGKC^2−19^ HNE may be considered as diagnostic biomarkers for RA. Importantly, elevated levels of IgM anti-HPT^78−108^ HNE, IgM anti-IGKC^2−19^, and IgG anti-THRB^328−345^ were positively correlated with the disease activity score in 28 joints for C-reactive protein (DAS28-CRP). Further, the ORs of RA development through IgM anti-HPT^78−108^ HNE (OR 5.235, *p* < 0.001), IgM anti-IGKC^2−19^ (OR 12.655, *p* < 0.001), and IgG anti-THRB^328−345^ (OR 5.761, *p* < 0.001) showed an increased risk. Lastly, we incorporated three machine learning models to differentiate RA from HC and OA, and performed feature selection to determine discriminative features. Experimental results showed that our proposed method achieved an area under the receiver operating characteristic curve of 0.92, which demonstrated that our selected autoantibodies combined with machine learning can efficiently detect RA.

**Conclusions:**

This study discovered that some IgG- and IgM-NAAs and anti-HNE M-NAAs may be correlated with inflammation and disease activity in RA. Moreover, our findings suggested that IgM anti-HPT^78−108^ HNE, IgM anti-IGKC^2−19^, and IgG anti-THRB^328−345^ may play heavy roles in RA development.

## Background

Rheumatoid arthritis (RA) is an autoimmune disorder with systemic inflammation and may be induced by oxidative stress that affects inflamed joints [1]. Age, sex, antinuclear antibodies, rheumatoid factor (RF), environmental factors and smoking have been investigated as risk factors of the RA etiopathogenesis [2–4]. Several autoantibodies against neoepitopes, including peptide modified with glycation, citrullination, carbamylation, and oxidation and malondialdehyde (MDA), have been measured in RA patients. In clinical practice, Anti-cyclic citrullinated peptide (anti-CCP) antibodies and RF are clinical biomarkers in patients with RA [5, 6]. One of the lipid peroxidation-generated highly bioactive electrophilic oxidation products, 4-hydroxy 2-nonenal (HNE), is produced through oxidative stress-generated reactive oxygen species (ROS)-targeted n-6 polyunsaturated fatty acids that contribute to the pathogenesis of various diseases, including RA, diabetes mellitus (DM), systemic lupus erythematosus (SLE), alcoholic liver disease (ALD), aging, neurodegenerative diseases, chronic obstructive pulmonary disease (COPD), cardiovascular diseases (CDs), and inflammation-driven cancers [7–10].

HNE has two reactive electrophilic groups, an aldehyde group and an alkene bond, and can react with residues in amino acid. The C = C double bond in HNE can be targeted via Michael addition and has a mass addition at 156 Da in its non-reduced form [alanine (A), arginine (R), cysteine (C), glutamine (Q), histidine (H), lysine (K) and leucine (L)] or 158 Da in its reduced form (CHKRQ) [11–13]. The aldehyde group in HNE can react by forming Schiff base adducts and increase mass of 138 Da in the non-reduced form (CHKAL) or 140 Da in the reduced form (CHKR) [11–13]. The non-reduced form of the Schiff base adducts (CHKR) further spontaneously rearranges to form a pyrrole adduct with a mass increase of 120 Da [14, 15].

Oxidation-specific epitopes (OSEs) include MDA, HNE, 2-(ω-carboxyethyl) pyrrole, oxidized phosphatidylserine, oxidized cardiolipin, oxidized phosphatidylethanolamine, and phosphocholine-oxidized phospholipids, which react with lipids or free amino groups in side chains of protein [5]. Eggleton et al*.* indicated that HNE-protein adducts present OSEs and are excellent immunogens to induce autoantibodies [8, 16]. Binder et al*.* revealed that chronic inflammation can be triggered by accumulation of OSEs [5]. Chou et al*.* suggested that many anti-OSEs are immunoglobulin M (IgM)-natural autoantibodies (IgM-NAAs) [17], and Gronwall et al*.* proposed that IgM-NAAs provide protection against pathogenesis of autoimmunity [18].

Levels of HNE-protein adducts in diseased states are higher than those in healthy controls (HCs), including Alzheimer's disease (AD), CDs, Menkes kinky hair disease (MKHD), hyperthyroidism, preeclampsia, mild cognitive impairment (MCI), RA, SLE, and breast cancer [7, 8, 19–25]. Luczaj et al*.* reported that amounts of HNE-protein adducts were significantly 1.21-fold greater in the plasma of patients with RA than levels in HCs [24]. Autoantibodies against HNE-derived epitopes are present in a variety of diseases comprising SLE, ALD, and AD [26–28]. However, to date, only few studies have reported autoantibodies against HNE-modified human serum albumin in RA [29].

In the present report, four differential novel HNE-modified peptides were re-analyzed via acquired tandem mass spectrometry (MS/MS) data (ProteomeXchange: PXD004546) using PEAKS 7 software (Bioinformatics Solutions, Waterloo, Canada) [6]. Acquired MS/MS spectra were obtained through concanavalin (Con) A affinity chromatography, one-dimensional (1-D) sodium dodecyl sulfate–polyacrylamide gel electrophoresis (SDS-PAGE), in-gel digestion, and nano-liquid chromatography tandem mass spectrometry (nano-LC–MS/MS) in patients with RA versus HCs [6]. We further validated HNE modifications of proteins and examined proteins level in serum and HNE-protein adducts. Moreover, we evaluated the performance of novel diagnostic autoantibodies against unmodified and HNE-modified peptides, which can possibly be used as diagnostic biomarkers for patients with RA, osteoarthritis (OA), and HCs. Herein, we aimed to determine correlations of IgM and IgG autoantibody titers against unmodified and HNE-modified peptide adducts with disease activity and clinical variables in RA patients. Further, the association between higher levels of serum autoantibodies in RA patients with a risk for RA development was assessed compared to HCs. Lastly, to thoroughly evaluate the potential of serum autoantibodies for biomarker development, we incorporated three machine learning algorithms and performed feature selection with WEKA (version 3.8.3) to further classify our subjects.

## Methods

### Patient samples

Serum samples from 155 female patients [60 with RA (54.8 ± 10.47 years old), 35 with OA (56.2 ± 11.44 years old), and 60 HCs (54.3 ± 8.70 years old)] were obtained from the Division of Allergy, Immunology, and Rheumatology, Department of Internal Medicine and the Department of Laboratory Medicine, Shuang-Ho Hospital (New Taipei City, Taiwan). Patients with RA had received a diagnosis from a rheumatologist and followed the appropriate criteria for classification—either the 2010 American College of Rheumatology (ACR)/European League Against Rheumatism classification criteria [30] or 1987 ACR classification criteria [31]. Further, patients with RA received a disease activity score in 28 joints for C-reactive protein (DAS28-CRP) (4.4 ± 1.67) assessment when they were diagnosed as RA. RA patients included in this study had suffered from this disease for a duration of 5.4 ± 6.41 years (Additional file 1: Table S1). OA patients had been diagnosed according to clinical symptoms with assistance from OA criteria by the ACR [32, 33]. Therapies were given to patients with OA (65% non-steroidal anti-inflammatory drugs (NSAIDs), 11.4% disease-modifying anti-rheumatic drugs (DMARDs)) and RA (42.9% NSAIDs, 99.3% DMARDs) by clinicians (Additional file 1: Table S1). The institutional review board of the study hospital approved this study, and informed consent was provided by all volunteers before participating. Four novel differential HNE-modified peptide adducts were re-identified using PEAKS 7 software (Bioinformatics Solutions) from previous MS/MS data (ProteomeXchange: PXD004546) [6]. Their protein levels were examined by Western blotting with individual randomly age paired serum from 32 patients with RA and 32 HCs. HNE modifications of HNE-modified peptide adducts were assessed through immunoprecipitation (IP) and Western blotting using the pooled Con A-captured serum samples from above-mentioned 32 pairs of serum samples. Further, serum levels of HNE-protein adducts were determined, and isotypes of autoantibodies against unmodified and HNE-modified peptides were evaluated among individual serum of 60 RA and 35 OA patients and 60 HCs. Clinical and demographic characteristics of patients with RA and OA and HCs are summarized in Additional file 1: Table S1. Serum was stored at -20 °C until analyzed.

### Novel differential HNE-modified peptide adducts were re-analyzed using PEAKS 7 software

The Peaks PTM module of PEAKS 7 software (Bioinformatics Solutions) was used to identify sequences of HNE-modified peptide from acquired MS/MS spectra against the Universal Protein Resource Knowledgebase, a human protein database (UniProt; http://www.uniprot.org/) containing 157,433 protein entities (UniProt, 2016/11), and those sequences are shown in Additional file 2: Figure S1A. MS/MS data are available through ProteomeXchange with the identifier PXD004546 [6]. S-Pyridylethylation (C)/ + 105.057849 Da was set as the fixed modification, whereas oxidation (M)/ + 15.994915 Da and the following HNE modifications were specified as variables: CHKRQAL/ + 156.11504 Da, CHKRQ/ + 158.13068 Da, CHKAL/ + 138.10446 Da, CHKR/ + 140.12012 Da, and CHKR/ + 120.1916 Da in Additional file 2: Figure S1B. All of the modified MS spectra were identified manually, and fragmented ions were labeled as y, b, y-NH_3_, and b-H_2_O ions. Details are provided in the "Additional file 3: Supplementary Information" section.

### Con A affinity chromatography and IP-Western blotting

Serum-derived Con A-captured serum proteins were purified using the protocol of Uen et al*.* [34]. Protein concentrations were examined using a Pierce™ Coomassie Plus (Bradford) Assay Kit (Thermo Scientific, Waltham, MA, USA) following to the protocol from manufacturer. Con A-captured proteins were used in IP. IP-Western blotting of pooled Con A-captured proteins was used to confirmed modifications of HNE-modified proteins. We used antibodies in IP including complement factor H (CFAH), haptoglobin (HPT), the Ig kappa chain C region (IGIK), and prothrombin (THRB), following to the protocol of Liao et al*.* [35]. HNE modifications of proteins were detected using a goat polyclonal anti-HNE antibody. Details of experiments are provided in the "Additional file 3: Supplementary Information" section.

### Detection of proteins and HNE-protein adducts

Levels of CFAH, HPT, IGKC, and THRB were detected using Western blotting. HNE-protein adducts were quantified using an enzyme-linked immunosorbent assay (ELISA) [36]. All samples were detected in duplicate. Details of the protocol are provided in the "Additional file 3: Supplementary Information" section.

### Measurement of autoantibodies against unmodified and HNE-modified peptides

Polypeptides were synthesized and used in the ELISA [35]. Unmodified peptides are presented as CFAH^1211−1230^, HPT^78−108^, IGKC^2−19^, and THRB^328−345^. HNE-modified peptides, marked as CFAH^1211−1230^ HNE, HPT^78−108^ HNE, IGKC^2−19^ HNE, and THRB^328−345^ HNE, were prepared using HNE (CAS 75899–68-2, Millipore, Darmstadt, Germany) [37]. Then, CFAH^1211−1230^ HNE and HPT^78−108^ HNE were reductively stabilized using NaBH_4_ [11]. In total, 155 serum samples were evaluated for the presence of IgG and IgM isotypes of anti-unmodified and anti-HNE-modified peptide autoantibodies. All of the samples were detected in duplicate. The protocol details are provided in the "Additional file 3: Supplementary Information" section.

### Statistical analysis

The significance of blot densitometric differences, and levels of serum proteins and HNE-protein adducts were determined using Student's *t*-test. A one-way analysis of variance (ANOVA) was used to examine levels of autoantibody isotypes against unmodified and HNE-modified peptides between RA and OA patients and HCs. Scheffe’s post-hoc test was applied to evaluate the difference of mean between any two groups, as well as a post-hoc test using the Bonferroni method with a 0.0167 adjusted significance level. We used GraphPad Prism (vers. 5.0; GraphPad Software, San Diego, CA, USA) to evaluate differences in Student's *t*-test between groups, correlations between measurements, and generated receiver operating characteristic (ROC) curves to evaluate the diagnostic performance of autoantibodies. Pearson’s or Spearman’s rank correlation coefficients were used to assess correlations among different parameters. To estimate multivariate-adjusted odds ratios (ORs) and their 95% confidence intervals (CIs) for RA risk, Logistic regression models were performed in this study. The positivity of autoantibody isotypes and HNE-protein adducts was decided by ROC curves. The cut-off value for an ROC curve was determined by Youden index, which represents the sum of sensitivity and 1-specificity, and the maximum value of Youden index is the suitable cut-off point for that curve. Pair-wise comparisons of ROC curves were assessed using MedCalc Statistical Software (vers. 15.4; MedCalc Software, Ostend, Belgium). One-way ANOVA and power were determined using SAS (vers. 9.3; SAS Institute, Cary, NC, USA), and power estimations were calculated according to the ROC analysis. The area under the ROC curve (AUC), sensitivity, and specificity were calculated at a 95% confidence level. The significance level of all statistical tests was set to *p* < 0.05. For feature selection, we first used ‘Information Gain’ as the attribute evaluator with ‘Ranker’ as the search method in WEKA (version 3.8) [38] to select discriminative features in identify RA patients. Next, we incorporated ten-fold cross-validation to evaluate our model based on decision trees (DT) [39], random forests [40], and support vector machines (SVM) [41] in scikit-learn (version 0.21.3) [42]. Parameter tuning was performed for each training and validation. During model selection, a forward selection algorithm was used to select the most effective combination of features for classification. In forward selection, a feature was selected in to the optimal feature set if adding the feature into the prediction model improved the AUC. To evaluate the predictive performance, we applied a confusion matrix to calculate the accuracy, precision, sensitivity, specificity, and AUC for assessments.

## Results

### Identification of differential HNE-modified peptide adducts

MS/MS spectra of the four HNE-modified peptides of patients were RA-specific as shown in Additional file 2: Figure S1 and Additional file 1: Table S1. HNE modifications were identified through manual examination. Among HNE-modified peptides, ^1211^-SHTLRTTCWDGKLEYPTCAK-^1230^ (CFAH^1211−1230^, Additional file 2: Figure S1C), ^78^-AVGDKLPECEADDGCPKPPEIAHGYVEH SVR-^108^ (HPT^78−108^, Additional file 2: Figure S1D), ^2^-TVAAPSVFIFPPSDEQLK-^19^ (IGKC^2−19^, Additional file 2: Figure S1E, upper panel), and ^328^-TFGSGEADCGLRPLFEKK-^345^ (THRB^328−345^, Additional file 2: Figure S1F) were identified. The HNE reduced form of Michael adducts of CFAH (at K1230) and HPT (at C92) corresponded to a mass increase of 158.13068 Da. The HNE Michael adducts of IGKC (at A5) and THRB (at K344) had a 156.11504 Da shift in the two residues. Moreover, four HNE-modified peptides that were HC-specific were shown in Additional file 2: Figure S1 and Additional file 1: Table S1: ^284−^ HRTGDEITYQCRNGFYPATRGNTAK^−308^ (CFAH^284−308^, Additional file 2: Figure S1G), ^162−^ILGGHLDAK^−170^ (HPT^162−170^. Additional file 2: Figure S1H), ^83−^VYACEVTHQGLSSPVTKSFNR^−103^ (IGKC^83−103^, Additional file 2: Figure S1I), and ^328−^TFGSGEADCGLRPLFEK^−344^ (THRB^328−344^, Additional file 2: Figure S1J) were identified. The HNE Schiff base adducts of CFAH (at K308) corresponded to a mass increase of 138.10446 Da. The HNE Michael adducts of HPT (at A169) and THRB (at L341) caused a 156.11504 Da shift in the two residues, and the HNE-reduced form of Michael adducts of THRB (at C336) corresponded to a mass increase of 140.12012 Da. The HNE-reduced form of Michael adducts of IGKC (at Q91) corresponded to a mass increase of 158.13068 Da. In particular, ^2^-TVAAPSVFIFPPSDEQLK-^19^ (IGKC^2−19^, Additional file 2: Figure S1E, bottom panel) was identified from RA and HC, respectively. The HNE Michael adducts of IGKC (at A4) corresponded to a mass increase of 156.11504 Da.

### Validation of HNE modifications on HNE-modified peptide adducts

**Fig. 1.**
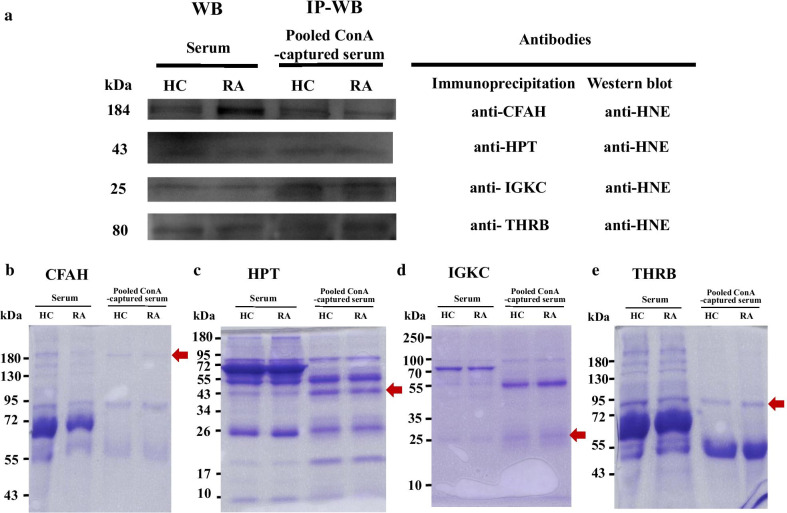
4-Hydroxy-2-nonenal (HNE) modification of proteins was validated using IP and Western blotting (**a**). Proteins were immunoprecipitated from pooled concanavalin (Con) A-captured serum samples (32 healthy controls (HCs) and 32 patients with rheumatoid arthritis (RA)) using anti-complement factor H (CFAH), anti-haptoglobin (HPT), anti-immunoglobulin kappa chain C region (IGKC), and anti-prothrombin (THRB) antibodies and then subjected to Western blotting with anti-HNE antibodies (upper panel). Individually selected random serum samples (HCs and RA patients) were used as controls; these were simultaneously used for Western blotting with anti-HNE antibodies. Percentages of the SDS-PAGE gel and IP loading amounts of Con A-captured serum proteins were 8% and 20 µg, 10% and 20 µg, 12% and 5 µg, and 8% and 20 µg for CFAH, HPT, IGKC, and THRB, respectively. A duplicate gel was stained with Coomassie brilliant blue (CBB) as a loading control, including CFAH (**b**), HPT (**c**), IGKC (**d**), and THRB (**e**). Red arrows indicate immunoprecipitated proteins

HNE modifications in four differential HNE-modified peptide adducts were validated by IP-Western blotting with two pooled Con A-captured serum samples from RA and HCs, which detected signals of approximately 184, 43, 25, and 80 kDa, respectively, indicating CFAH, HPT, IGKC, and THRB (Fig. 1).

### Detection of protein levels and HNE-protein adduct levels

**Fig. 2:**
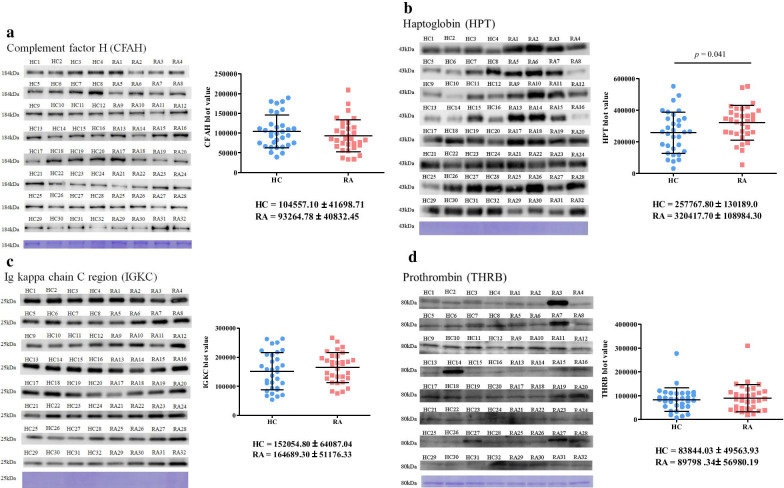
Protein levels of complement factor H (CFAH), haptoglobin (HPT), immunoglobulin kappa chain C region (IGKC), and prothrombin (THRB) in serum were respectively examined using anti-CFAH (**a**), anti-HPT (**b**), anti-IGKC (**c**), and anti-THRB (**d**) antibodies through Western blotting. Average densitometric values were calculated from duplicate data. Percentages of the SDS-PAGE gel and loading amounts of serum proteins used in Western blotting were 8% and 20 µg, 10% and 5 µg, 12% and 1 µg, and 8% and 20 µg for CFAH, HPT, IGKC, and THRB, respectively. Membrane Coomassie brilliant blue (CBB) staining was used as a loading control (bottom panel). Student’s *t*-test was used to determine the significance of blot densitometric differences, levels of serum proteins, and HNE-protein adducts. Values of mean ± 1 standard deviation are also indicated in the figure as horizontal lines

Protein levels of CFAH, IGKC, and THRB from 32 randomly paired individual serum samples from RA patients and HCs showed no significant differences (Fig. 2a, c, d). However, levels of HPT in patients with RA were greatly higher than the levels in HCs (1.24-fold, *p* < 0.041, Fig. 2b). Serum levels of HNE-protein adducts in RA patients were significantly higher than the levels in OA patients (1.16-fold, *p* = 0.0311) and HCs (1.20-fold, *p* = 0.0062, Additional file 1: Table S1).

### Measuring autoantibodies against unmodified and HNE-modified peptides

**Fig. 3:**
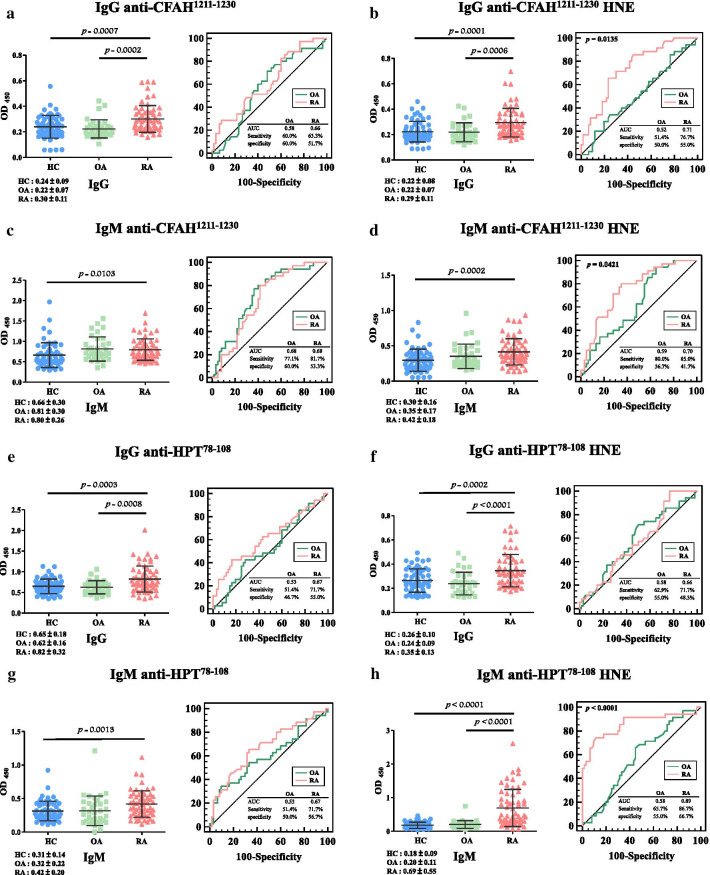

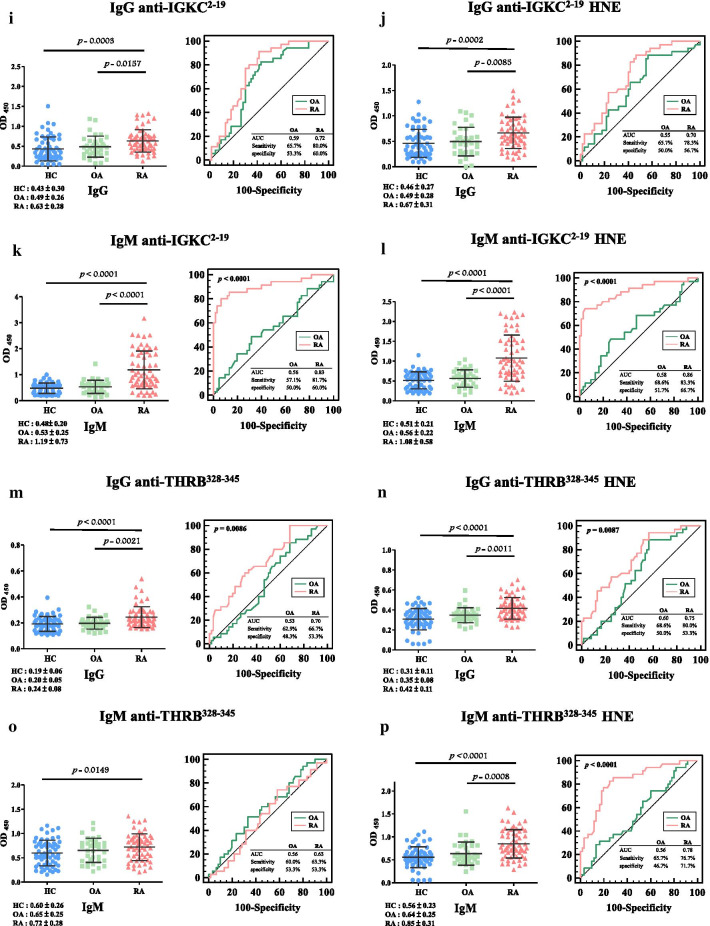
Dot plots and receiver operating characteristics (ROC) curves of serum concentrations (absorbance units at 450 nm) of autoantibody isotypes: IgG anti-complement factor H (CFAH)^1211–1230^ (**a**), IgG anti-CFAH^1211−1230^ 4-hydroxy-2-nonenal (HNE) (**b**), IgM anti-CFAH^1211−1230^ (**c**), IgM anti-CFAH^1211−1230^ HNE (**d**), IgG anti-haptoglobin (HPT)^78–108^ (**e**), IgG anti-HPT^78−108^ HNE (**f**), IgM anti-HPT^78−108^ (**g**), IgM anti-HPT^78−108^ HNE (**h**), IgG anti-immunoglobulin kappa chain C region (IGKC)^2–19^ (**i**), IgG anti-IGKC^2−19^ HNE (**j**), IgM anti-IGKC^2−19^ (**k**), IgM anti-IGKC^2−19^ HNE (**l**), IgG anti-prothrombin (THRB)^328–345^ (**m**), IgG anti-THRB^328−345^ HNE (**n**), IgM anti-THRB^328−345^ (**o**), and IgM anti-THRB^328−345^ HNE (**p**) in healthy controls (HCs), osteoarthritis (OA) patients, and rheumatoid arthritis (RA) patients using an ELISA. OD_450_, optical density at 450 nm. ANOVA was used to test levels of autoantibody isotypes among patients with RA and OA, and HCs. Scheffe’s post-hoc test was applied to compare the mean difference between any two groups. Values of mean ± 1 standard deviation are also indicated in the figure as horizontal lines

The AUC, sensitivity, and specificity were used to assess clinical performances of IgG and IgM that against unmodified and HNE-modified peptides. The ANOVA analysis indicated that differences in all autoantibodies against unmodified and HNE-modified peptides were significant among patients with RA, OA, and HCs (Fig. 3, Table 1).

**Table 1:** Differential 4-hydroxy-2-nonenal (HNE)-modified peptide adducts in rheumatoid arthritis (RA) patients compared to healthy controls (HCs)

Protein^a^	Modified peptide^b^	Position^c^	Obs.^d^	Calc.^e^	dM^f^	dM^g^	Sequence foundwith modifications	
							HC	RA
CFAH	SHTLRTTCWDGKLEYPTCAK(+ 158)	1211–1230	858.439	858.435	0.0041	5.4	−	+
	HRTGDEITYQCRNGFYPATRGNTAK(+ 138)	284–308	749.370	749.372	− 0.0018	−1.8	+	−
HPT	AVGDKLPECEADDGC(+ 158)PKPPEIAHGYVEHSVR	78–108	869.925	869.927	− 0.0017	−1.3	−	+
	ILGGHLDA(+ 156)K	162–170	540.329	540.327	0.0028	6.1	+	−
IGKC	TVAA(+ 156)PSVFIFPPSDEQLK	2–19	701.384	701.386	− 0.0018	−1.7	−	+
	TVA(+ 156)APSVFIFPPSDEQLK	2–19	1051.582	1051.575	0.0070	7.1	+	+
	VYACEVTHQ(+ 158)GLSSPVTKSFNR	83–103	862.780	862.785	− 0.0044	−4.5	+	−
THRB	TFGSGEADCGLRPLFEK(+ 156)K	328–345	1056.036	1056.046	− 0.0107	−9.6	−	+
	TFGSGEADC(+ 140)GLRPL(+ 156)FEK	328–344	708.376	708.375	0.0009	2.1	+	−

^a^CFAH, Complement factor H; HPT, Haptoglobin; IGKC, Ig kappa chain C region; THRB, Prothrombin

^b^Site of modified peptide

^c^Amino acid positions of the first and the last residues in peptide

^d^Obs.: observed m/z of the modified peptides

^e^Calc.: calculated m/z of the modified peptides

^f^dM (Obs.-Calc.): mass accuracy

^g^Modified peptide mass accuracy (ppm)

Levels of IgG against CFAH^1211−1230^ in patients with RA were greatly higher than those in patients with OA (1.36-fold, *p* = 0.0002) and HCs (1.25-fold, *p* = 0.0007) (Fig. 3a, left panel), and AUC values were 0.66 (with 63.3% sensitivity and 51.7% specificity), and 0.58 (with 60.0% sensitivity and 60.0% specificity) for detecting patients with RA and OA (Table 2). Levels of IgG against CFAH^1211−1230^ HNE in RA patients were greatly higher than the levels in OA patients by 1.32-fold (*p* = 0.0006), and HCs by 1.32-fold higher (*p* = 0.0001) (Fig. 3b, left panel), and AUC values were 0.71 (with 76.7% sensitivity and 55.0% specificity) and 0.52 (with 51.4% sensitivity and 50.0% specificity) for detecting patients with RA and OA (Table 2). HC-RA versus HC-OA showed a statistically significant difference (*p* = 0.0135, Fig. 3b, right panel, Table 2) in pair-wise comparisons of ROC curves. Further, levels of IgM against CFAH^1211−1230^ in RA patients were greatly higher than the levels in HCs by 1.21-fold (*p* = 0.0103) (Fig. 3c, left panel), and AUC values were 0.68 (with 81.7% sensitivity and 53.3% specificity) and 0.68 (with 77.1% sensitivity and 60.0% specificity) for detecting patients with RA and OA (Table 2). Levels of IgM against CFAH^1211−1230^ HNE in RA patients were greatly higher than the levels in HCs by 1.40-fold (*p* = 0.0002, Fig. 3d, left panel), and AUC values were 0.70 (with 85.0% sensitivity and 41.7% specificity) and 0.59 (with 80.0% sensitivity and 36.7% specificity) for detecting patients with OA and RA (Table 2). Further, HC-OA versus HC-RA showed a significant difference (*p* = 0.0421, Fig. 3d, right panel, Table 2) in pair-wise comparisons of ROC curves.

**Table 2:** Comparisons of the area under the receiver operating characteristic curve (AUC), sensitivity, and specificity of autoantibody isotypes against unmodified and 4-hydroxy-2-nonenal (HNE)-modified peptides in rheumatoid arthritis (RA) patients and osteoarthritis (OA) patients compared to healthy controls (HCs)

Peptides	RA vs*.* HC			OA versus HC			*p* value ^a^
	AUC (95%CI)	Sensitivity (95%CI)	Specificity (95%CI)	AUC (95%CI)	Sensitivity (95%CI)	Specificity (95%CI)	
IgG anti-CFAH^1121−1230^	0.66 (0.57–0.76)	63.3% (49.9–75.4)	51.7% (38.4–64.8)	0.58 (0.46–0.70)	60.0% (42.1–76.1)	60.0% (46.5–72.4)	0.6844
Cutoff (OD_450_)		> 0.24			< 0.21		
IgG anti-CFAH^1121−1230^ HNE	0.71 (0.62–0.80)	76.7% (64.0–86.6)	55.0% (41.6–67.9)	0.52 (0.40–0.64)	51.4% (34.0–68.6)	50.0% (36.8–63.2)	0.0135
Cutoff (OD_450_)		> 0.21			< 0.20		
IgM anti-CFAH^1121−1230^	0.68 (0.59–0.78)	81.7% (69.6–90.5)	53.3% (40.0–66.3)	0.68 (0.58–0.79)	77.1% (59.9–89.6)	60.0% (46.5–72.4)	0.4816
Cutoff (OD_450_)		> 0.58			> 0.63		
IgM anti-CFAH^1121−1230^ HNE	0.70 (0.61–0.79)	85.0% (73.4–92.9)	41.7% (29.1–55.1)	0.59 (0.47–0.71)	80.0% (63.1–91.6)	36.7% (24.6–50.1)	0.0421
Cutoff (OD_450_)		> 0.24			> 0.23		
IgG anti-HPT^78−108^	0.67 (0.58–0.77)	71.7% (58.6–82.6)	55.0% (41.6–67.9)	0.53 (0.41–0.65)	51.4% (34.0–68.6)	46.7% (33.7–60.0)	0.3711
Cutoff (OD_450_)		> 0.64			< 0.64		
IgG anti-HPT^78−108^ HNE	0.66 (0.57–0.76)	71.7% (58.6–82.6)	48.3% (35.2–61.6)	0.58 (0.46–0.70)	62.9% (44.9–78.5)	55.0% (41.6–67.9)	0.8974
Cutoff (OD_450_)		> 0.25			< 0.25		
IgM anti-HPT^78−108^	0.67 (0.57–0.77)	71.7% (58.6–82.6)	56.7% (43.2–69.4)	0.53(0.40–0.66)	51.4%(33.9–68.6)	50.0%(36.8– 63.2)	0.2823
Cutoff (OD_450_)		> 0.30			< 0.29		
IgM anti-HPT^78−108^ HNE	0.89 (0.80–0.93)	86.7% (75.4–94.1)	66.7% (53.3–78.3)	0.58 (0.46–0.69)	65.7% (47.8–80.9)	55.0% (41.6–67.9)	< 0.0001
Cutoff (OD_450_)		> 0.20			> 0.16		
IgG anti-IGKC^2−19^	0.72 (0.62–0.81)	80.0% (67.7–89.2)	60.0% (46.5–72.4)	0.59 (0.47–0.70)	65.7% (47.8–80.9)	53.3% (40.0–66.3)	0.1471
Cutoff (OD_450_)		> 0.41			> 0.37		
IgG anti-IGKC^2−19^ HNE	0.70 (0.61–0.79)	78.3% (65.8–87.9)	56.7% (43.2–69.4)	0.55 (0.43–0.67)	65.7% (47.9–80.9)	50.0% (36.8–63.2)	0.0917
Cutoff (OD_450_)		> 0.45			> 0.39		
IgM anti-IGKC^2−19^	0.83 (0.75–0.90)	81.7% (69.6–90.5)	60.0% (46.5–72.4)	0.56 (0.44–0.68)	57.1% (39.4–73.7)	50.0% (36.8–63.2)	< 0.0001
Cutoff (OD_450_)		> 0.61			> 0.53		
IgM anti-IGKC^2−19^ HNE	0.86 (0.79–0.93)	83.3% (71.5–91.7)	66.7% (53.3–78.3)	0.58 (0.46–0.70)	68.6% (50.7–83.2)	51.7% (38.4–64.8)	< 0.0001
Cutoff (OD_450_)		> 0.37			> 0.32		
IgG anti-THRB^328−345^	0.70 (0.61–0.79)	66.7% (53.3–78.3)	53.3% (40.0–66.3)	0.53 (0.42–0.65)	62.9% (44.9–78.5)	48.3% (35.2–61.6)	0.0086
Cutoff (OD_450_)		> 0.20			> 0.19		
IgG anti-THRB^328−345^ HNE	0.75 (0.67–0.84)	80.0% (67.7–89.2)	53.3% (40.0–66.3)	0.60 (0.48–0.71)	68.6% (50.7–83.2)	50.0% (36.8–63.2)	0.0087
Cutoff (OD_450_)		> 0.32			> 0.31		
IgM anti-THRB^328−345^	0.63 (0.53–0.73)	63.3% (50.0–75.4)	53.3% (40.0–66.3)	0.56 (0.44–0.68)	60.0% (42.1–76.1)	53.3% (40.0–66.3)	0.5845
Cutoff (OD_450_)		> 0.61			> 0.60		
IgM anti-THRB^328−345^ HNE	0.78 (0.69–0.86)	76.7% (64.0–86.6)	71.7% (58.6–82.6)	0.56 (0.44–0.68)	65.7% (47.8–80.9)	46.7% (33.7–60.0)	0.0001
Cutoff (OD_450_)		> 0.64			> 0.52		

^a^Pairwise comparison of predictive performance, HC-RA versus HC-OA, was performed by Student’s *t*-test

Levels of IgG against HPT^78−108^ were significantly 1.32- (*p* = 0.0008) and 1.26-fold (*p* = 0.0003) greater in patients with RA than the levels in OA patients and HCs (Fig. 3e, left panel), and AUC values were 0.67 (with 71.7% sensitivity and 55.0% specificity) and 0.53 (with 51.4% sensitivity and with 46.7% specificity) for detecting patients with RA and OA (Table 2). Levels of anti-HPT^78−108^ HNE IgG in RA patients were greatly higher than the levels in OA patients and HCs by 1.46- (*p* < 0.0001) and 1.35-fold (*p* = 0.0002), respectively (Fig. 3f, left panel), and AUC values were 0.66 (with 71.7% sensitivity and 48.3% specificity) and 0.58 (with 62.9% sensitivity and 55.0% specificity) for detecting patients with RA and OA (Table 2). Levels of IgM against HPT^78−108^ were 1.35-fold (*p* = 0.0013) greatly higher in RA patients than HCs (Fig. 3g, left panel), and AUC values were 0.67 (with 71.7% sensitivity and 56.7% specificity) for detecting RA and 0.53 (with 51.4% sensitivity and 50.0% specificity) for detecting OA (Table 2). Levels of anti-HPT^78−108^ HNE IgM in RA patients were greatly higher than the levels in OA patients by 3.45-fold (*p* < 0.0001), while RA patients versus HCs was 3.83-fold higher (*p* < 0.0001) (Fig. 3h, left panel), and AUC values were 0.89 (with 86.7% sensitivity and 66.7% specificity) for detecting RA and 0.58 (with 65.7% sensitivity and 55.0% specificity) for detecting OA (Table 2). Further, HC-RA and HC-OA were statistically significantly different (*p* < 0.0001, Fig. 3h, right panel, Table 2) in pair-wise comparisons of ROC curves.

Levels of IgG against IGKC^2−19^ were greatly higher in patients with RA than in patients with OA (1.29-fold, *p* = 0.0157) and HCs (1.47-fold, *p* = 0.0003) (Fig. 3i, left panel), and AUC values were 0.72 (with 80.0% sensitivity and 60.0% specificity) for detecting RA and 0.59 (with 65.7% sensitivity and 53.3% specificity) for detecting OA (Table 2). Levels of IgG against IGKC^2−19^ HNE in RA patients were greatly higher than the levels in OA patients by 1.37-fold (*p* = 0.0085) and HCs by 1.46-fold (*p* = 0.0002) (Fig. 3j, left panel), and AUC values were 0.70 (with 78.3% sensitivity and 56.7% specificity) for detecting RA and 0.55 (with 65.7% sensitivity and 50.0% specificity) for detecting OA (Table 2). Levels of IgM against IGKC^2−19^ in RA patients were greatly higher than the levels in OA patients by 2.24-fold (*p* < 0.0001) and HCs by 2.48-fold (*p* < 0.0001) (Fig. 3k, left panel), and AUC values were 0.83 (with 81.7% sensitivity and 60.0% specificity) for RA detection and 0.56 (with 57.1% sensitivity and 50.0% specificity) for detecting OA (Table 2). HC-RA versus HC-OA was statistically significant (*p* < 0.0001, Fig. 3k, right panel, Table 2), in pair-wise comparisons of ROC curves. Levels of IgM against IGKC^2−19^ HNE were significantly 1.93- (*p* < 0.0001) and 2.12-fold (*p* < 0.0001) greater in RA patients than in OA patients and HCs, respectively (Fig. 3l, left panel), and AUC values were 0.86 (with 83.3% sensitivity and 66.7% specificity) for detecting RA and 0.58 (with 68.6% sensitivity and 51.7% specificity) for detecting OA (Table 2). Further, HC-RA versus HC-OA was statistically significant (*p* < 0.0001, Fig. 3l, right panel, Table 2) in pair-wise comparisons of ROC curves.

Levels of IgG against THRB^328−345^ were greatly higher in RA patients than OA patients (1.20-fold, *p* = 0.0021) and HCs (1.26-fold, *p* < 0.0001) (Fig. 3m, left panel), and AUC values were 0.70 (with 66.7% sensitivity and 53.3% specificity) for detecting RA and 0.53 (with 62.9% sensitivity and 48.3% specificity) for detecting OA (Table 2). Thus, HC-RA versus HC-OA statistically significantly differed (*p* = 0.0086, Fig. 3m, right panel, Table 2) in pair-wise comparisons of ROC curves. Levels of IgG against THRB^328−345^ HNE were significantly 1.20- (*p* = 0.0011) and 1.35-fold (*p* < 0.0001) greater in RA patients than in OA patients and HCs (Fig. 3n, left panel), respectively, and AUC values were 0.75 (with 80.0% sensitivity and 53.3% specificity) and 0.60 (with 68.6% sensitivity and 50.0% specificity) for detecting RA and OA (Table 2). HC-RA versus HC-OA was statistically significant (*p* = 0.0087, Fig. 3n, right panel, Table 2) in pair-wise comparisons of ROC curves. Further, levels of IgM antibodies against anti-THRB^328−345^ in RA patients was greatly higher than HCs by 1.20-fold (*p* = 0.0149, Fig. 3o, left panel), and AUC values were 0.63 (with 63.3% sensitivity and 53.3% specificity) and 0.56 (with 60.0% sensitivity and 53.3% specificity) for detecting RA and OA (Table 2). Levels of IgM against anti-THRB^328−345^ HNE were 1.33- (*p* = 0.0008) and 1.52-fold (*p* < 0.0001) greater in RA patients than in OA patients and HCs, respectively (Fig. 3p, left panel), and AUC values were 0.78 (with 76.7% sensitivity and 71.7% specificity) and 0.56 (with 65.7% sensitivity and 46.7% specificity) for detecting RA and OA (Table 2). HC-RA versus HC-OA was statistically significant (*p* < 0.0001, Fig. 3p, right panel, Table 2) in pair-wise comparisons of ROC curves.

### Correlations of serum anti-unmodified and anti-HNE-modified peptide autoantibodies with clinical variables in RA patients

A correlation analysis was conducted of autoantibody reactivities against unmodified and HNE-modified peptides with DAS28-CRP measurements and serum clinical variables (RF, anti-CCP, CRP, ESR, and the HNE-protein adduct) in patients with RA. In Table 3, there were significant positive correlations between DAS28-CRP scores and autoantibodies, including IgM anti-HPT^78−108^ HNE (*r* = 0.2703,* p* = 0.0367), IgM anti-IGKC^2−19^ (*r* = 0.2816, *p* = 0.0293), and IgG anti-THRB^328−345^ (*r* = 0.2703, *p* = 0.0367). RF vs*.* autoantibodies exhibited significant positive correlations, including IgM anti-HPT^78−108^ HNE (*r* = 0.6140, *p* < 0.0001), IgM anti-IGKC^2−19^ (*r* = 0.5674, *p* < 0.0001), IgM anti-IGKC^2−19^ HNE (*r* = 0.5404, *p* < 0.0001), IgG anti-THRB^328−345^ (*r* = 0.614, *p* < 0.0001), IgG anti-THRB^328−345^ HNE (*r* = 0.3072, *p* = 0.019), and IgM anti-THRB^328−345^ HNE (*r* = 0.2845, *p* = 0.0304). Anti-CCP versus autoantibodies exhibited significant positive correlations, including IgG anti-HPT^78−108^ HNE (*r* = 0.2782, *p* = 0.0314) and IgG anti-THRB^328−345^ HNE (*r* = 0.2549, *p* = 0.0494). ESR vs*.* autoantibodies exhibited significant positive correlations, including IgM anti-IGKC^2−19^ (*r* = 0.2692, *p* = 0.0376), IgM anti-IGKC^2−19^ HNE (*r* = 0.2985, *p* = 0.0205), and IgM anti-THRB^328−345^ HNE (r = 0.2597, *p* = 0.0451). Moreover, HNE-protein adduct vs*.* autoantibodies exhibited significant positive correlations, including IgM anti-IGKC^2−19^ (*r* = 0.2667, *p* = 0.0394) and IgM anti-IGKC^2−19^ HNE (*r* = 0.2709, *p* = 0.0363). However, autoantibodies exhibited significant negative correlations between DAS28-CRP scores, including IgG anti-IGKC^2−19^ (*r* = − 0.3538, *p* = 0.0056) and IgG anti-IGKC^2−19^ HNE (*r* = − 0.3432, *p* = 0.0073). HNE-protein adducts vs*.* IgM anti-THRB^328−345^ (*r* = − 0.2796, *p* = 0.0305) had a significantly negative correlation. Otherwise, there were no significant correlations between CRP and the other autoantibodies (Table 3).

**Table 3:** Correlations between autoantibody isotypes against unmodified and 4-hydroxy-2-nonenal (HNE)-modified peptides and clinical variables in rheumatoid arthritis (RA) patients (*n* = 60)

	DAS28		RF		Anti-CCP		CRP		ESR		HNE adduct	
	r	*p* value	r	*p* value	r	*p* value	r	*p* value	r	*p* value	r	*p* value
IgG anti-CFAH^1121−1230^	0.1580	0.2279	0.0968	0.4696	0.0108	0.9347	0.0229	0.8659	0.2076	0.1115	− 0.009	0.9458
IgG anti-CFAH^1121−1230^ HNE	− 0.0441	0.7379	− 0.0035	0.9794	− 0.0359	0.7852	− 0.1446	0.2831	0.1359	0.3006	− 0.0082	0.9501
IgM anti-CFAH^1121−1230^	0.0424	0.7477	0.0626	0.6406	0.2100	0.1073	− 0.0164	0.9036	0.0756	0.5658	− 0.0503	0.7026
IgM anti-CFAH^1121−1230^HNE	0.0377	0.7748	0.0435	0.746	0.2192	0.0924	− 0.0821	0.5435	0.1343	0.3063	0.0302	0.8186
IgG anti-HPT^78−108^	0.0929	0.4804	0.1386	0.2996	0.0659	0.6169	0.1138	0.3994	0.1695	0.1954	0.0754	0.5670
IgG anti-HPT^78−108^ HNE	− 0.0266	0.8400	0.0195	0.8848	0.2782	0.0314	0.1081	0.4236	0.1346	0.3052	− 0.0593	0.6529
IgM anti-HPT^78−108^	− 0.0171	0.8969	0.1711	0.1992	0.0877	0.5051	− 0.0591	0.6623	0.0074	0.9555	− 0.0453	0.7310
IgM anti-HPT^78−108^ HNE	0.2703	0.0367	0.6140	< 0.0001	0.0294	0.8238	0.1236	0.3596	0.2180	0.0943	0.1836	0.1603
IgG anti-IGKC^2−19^	− 0.3538	0.0056	− 0.1144	0.3926	− 0.0023	0.9862	− 0.1061	0.4320	− 0.123	0.3597	0.2097	0.1078
IgG anti-IGKC^2−19^ HNE	0.6023	− 0.3432	0.0073	− 0.1997	0.1328	0.1253	0.3399	− 0.0524	0.6986	− 0.0328	0.8035	0.0686
IgM anti-IGKC^2−19^	0.2816	0.0293	0.5674	< 0.0001	− 0.0588	0.6553	0.0511	0.7059	0.2692	0.0376	0.2667	0.0394
IgM anti-IGKC^2−19^ HNE	0.2487	0.0553	0.5404	< 0.0001	0.2439	0.0604	0.0429	0.7512	0.2985	0.0205	0.2709	0.0363
IgG anti-THRB^328−345^	0.2703	0.0367	0.6140	< 0.0001	0.2439	0.0604	0.1236	0.3596	0.218	0.0943	0.1836	0.1603
IgG anti-THRB^328−345^ HINE	0.1274	0.3320	0.3072	0.019	0.2549	0.0494	− 0.0575	0.6712	0.1341	0.307	0.0170	0.8972
IgM anti-THRB^328−345^	− 0.0637	0.6290	0.2207	0.096	− 0.0365	0.7821	− 0.0292	0.8293	− 0.1513	0.2473	− 0.2796	0.0305
IgM anti-THRB^328−345^ HNE	0.0818	0.5345	0.2845	0.0304	− 0.0438	0.7397	0.081	0.549	0.2597	0.0451	0.1036	0.4310

### Associations of serum anti-unmodified and anti-HNE-modified peptide autoantibodies with RA patients compared to HCs

As shown in Table 4, an age adjusted logistic regression analysis demonstrated that ORs of RA development were significantly associated with levels of autoantibodies against unmodified and HNE-modified peptides in patients with RA compared to HCs: IgG anti-CFAH^1121−1230^ (OR 3.293, *p* = 0.013, power = 0.874), IgG anti-CFAH^1121−1230^ HNE (OR 2.808, *p* = 0.005, power = 0.870), IgM anti-CFAH^1121−1230^ (OR 5.204, *p* < 0.001, power = 0.979), IgM anti-CFAH^1121−1230^ HNE (OR 2.700, *p* = 0.004, power = 0.838), IgM anti-HPT^78−108^ (OR = 2.695, *p* = 0.005, power = 0.841), IgM anti-HPT^78−108^ HNE (OR 5.235, *p* < 0.001, power = 0.985), IgG anti-IGKC^2−19^ (OR 4.679, *p* < 0.001, power = 0.974), IgM anti-IGKC^2−19^ HNE (OR 3.206, *p* < 0.001, power = 0.905), IgM anti-IGKC^2−19^ (OR 12.655, *p* < 0.001, power > 0.999), IgM anti-IGKC^2−19^ HNE (OR 8.095, *p* < 0.001, power > 0.999), IgG anti-THRB^328−345^ (OR 5.761, *p* < 0.001, power = 0.951), IgG anti-THRB^328−345^ HNE (OR 9.524, *p* < 0.001, power = 0.962), and IgM anti-THRB^328−345^ HNE (OR 5.043, *p* < 0.001, power = 0.992). IgM anti-IGKC^2−19^ carried the highest risk of RA (Table 4). The OR results were not considered because of power values less than 0.7, including IgG anti-HPT^78−108^, IgG anti-HPT^78−108^ HNE, and IgM anti-THRB^328−345^. Further, HNE-protein adducts (OR 2.413, *p* = 0.014, power = 0.743) also demonstrated a high risk of RA development (Table 4).

**Table 4:** Associations of 4-hydroxy-2-nonenal (HNE)-protein adducts and autoantibody isotypes against unmodified and HNE-modified peptides in rheumatoid arthritis (RA) patients vs*.* healthy controls (HCs)

Risk factor		Cutoff	HC	RA	Age-adjusted logistic regression model^a^	*p* value	Power
			(n)	(n)	OR (95% CI)		
HNE-protein adduct	<	2.386	26	24	1.0 (Ref)	0.014	0.743
	≥	2.386	34	71	2.413 (1.193, 4.880)		
IgG anti-CFAH^1121−1230^	<	0.175	14	8	1.0 (Ref)	0.013	0.874
	≥	0.175	46	87	3.293 (1.286, 8.431)		
IgG anti-CFAH^1121−1230^ HNE	<	0.263	46	51	1.0 (Ref)	0.005	0.870
	≥	0.263	14	44	2.808 (1.363, 5.785)		
IgM anti-CFAH^1121−1230^	<	0.609	35	20	1.0 (Ref)	< 0.001	0.979
	≥	0.609	25	75	5.204 (2.550, 10.617)		
IgM anti-CFAH^1121−1230^ HNE	<	0.333	39	39	1.0 (Ref)	0.004	0.838
	≥	0.333	21	56	2.700 (1.378, 5.289)		
IgG anti-HPT^78−108^	<	0.662	35	37	1.0 (Ref)	0.024	0.645
	≥	0.662	25	58	2.156 (1.108, 4.193)		
IgG anti-HPT^78−108^ HNE	<	0.307	41	51	1.0 (Ref)	0.094	0.455
	≥	0.307	19	44	1.821 (0.904, 3.672)		
IgM anti-HPT^78−108^	<	0.326	40	42	1.0 (Ref)	0.005	0.841
	≥	0.326	20	53	2.695 (1.356, 5.354)		
IgM anti-HPT^78−108^ HNE	<	0.187	39	25	1.0 (Ref)	< 0.001	0.985
	≥	0.187	21	70	5.235 (2.594, 10.565)		
IgG anti-IGKC^2−19^	<	0.392	35	22	1.0 (Ref)	< 0.001	0.974
	≥	0.392	25	73	4.679 (2.317, 9.449)		
IgG anti-IGKC^2−19^ HNE	<	0.427	33	26	1.0 (Ref)	< 0.001	0.905
	≥	0.427	27	69	3.206 (1.621, 6.340)		
IgM anti-IGKC^2−19^	<	0.743	56	50	1.0 (Ref)	< 0.001	> 0.999
	≥	0.743	4	45	12.655 (4.244, 37.734)		
IgM anti-IGKC^2−19^ HNE	<	0.738	53	47	1.0 (Ref)	< 0.001	> 0.999
	≥	0.738	7	48	8.095 (3.312, 19.780)		
IgG anti-THRB^328−345^	<	0.154	19	7	1.0 (Ref)	< 0.001	0.951
	≥	0.154	41	88	5.761 (2.228, 14.895)		
IgG anti-THRB^328−345^ HNE	<	0.281	26	7	1.0 (Ref)	< 0.001	0.962
	≥	0.281	34	88	9.524 (3.777, 24.012)		
IgM anti-THRB^328−345^	<	0.791	46	56	1.0 (Ref)	0.029	0.697
	≥	0.791	14	39	2.249 (1.086, 4.659)		
IgM anti-THRB^328−345^ HNE	<	0.682	48	42	1.0 (Ref)	< 0.001	0.992
	≥	0.682	12	53	5.043 (2.377, 10.696)		

^a^OR, odds ratio; Ref, reference value

### Using serum anti-unmodified and anti-HNE-modified peptide autoantibodies to identify RA patients from HCs and OA patients

Experimental results from feature selection indicated that IgM anti-HPT^78−108^ HNE (HC vs. RA 0.3496, OA vs. RA 0.3496), IgM anti-IGKC^2−19^(HC vs. RA 0.2967), and IgM anti-IGKC^2−19^ HNE (HC vs. RA 0.2921) showed discriminative power in identifying RA patients from HC and OA patients (Table 5). Predictive performance of decision trees, random forests, and support vector machines based on all 16 autoantibodies and only forward-selected autoantibodies were summarized in Tables 6 and 7, respectively . For both groups (HC vs. RA and OA vs. RA), we observed that using only forward-selected autoantibodies consistently performed better than all autoantibodies, which supports our assumption that feature selection is effective to identify RA. For HC versus RA, the decision tree achieved an AUC of 0.86, random forest achieved AUC of 0.92, and support vector machine achieved an AUC of 0.82. For OA versus RA, decision tree achieved an AUC of 0.84, random forest achieved an AUC of 0.92, and support vector machine achieved an AUC of 0.88. The ROC plots of these algorithms were presented in Additional file 4: Figure S2. Our results demonstrated that random forest performed better than the other algorithms for predicting RA from HC or OA.

**Table 5:** Feature importance ranking of 4-hydroxy-2-nonenal (HNE)-protein adducts and autoantibody isotypes against unmodified and HNE-modified peptides in rheumatoid arthritis (RA) patients versus healthy controls (HCs) and osteoarthritis (OA) patients

HC versus RA		OA versus RA	
Score	Feature	Score	Feature
0.3496	IgM anti-HPT^78−108^ HNE	0.352	IgM anti-HPT^78−108^ HNE
0.2967	IgM anti-IGKC^2−19^	0.245	IgM anti-IGKC^2−19^
0.2921	IgM anti-IGKC^2−19^ HNE	0.223	IgM anti-IGKC^2−19^ HNE
0.1689	IgM anti-THRB HNE	0.186	IgG anti-HPT^78−108^ HNE
0.1395	IgG anti-THRB^328−345^	0.172	IgG anti-CFAH^1121−1230^
0.1382	IgG anti-THRB^328−345^ HNE	0.139	HNE adducts
0.1312	IgG anti-HPT^78−108^ HNE	0.134	IgM anti-THRB^328−345^ HNE
0.1186	IgG anti-IGKC^2−19^	0.128	IgG anti-CFAH^1121−1230^ HNE
0.1123	IgG anti-CFAH^1121−1230^	0.126	IgG anti-HPT^78−108^
0.1108	IgM anti-CFAH^1121−1230^	0.114	IgG anti-THRB^328−345^
0.1007	IgG anti-HPT^78−108^		
0.0994	HNE adducts		
0.0980	IgG anti-CFAH^1121−1230^ HNE

**Table 6:** Predictive performance using all 16 features in decision trees, random forests, and support vector machines for healthy controls (HCs) versus rheumatoid arthritis (RA) patients and osteoarthritis (OA) versus RA patients

Algorithms*	Accuracy	Precision	F1 score	Sensitivity	Specificity	AUC
HC v.s. RA						
DT	0.75 (0.70–0.79)	0.77 (0.69–0.86)	0.69 (0.63–0.75)	0.62 (0.56–0.69)	0.85 (0.81–0.90)	0.74 (0.69–0.78)
RF	0.80 (0.79–0.82)	0.83 (0.81–0.85)	0.79 (0.77–0.80)	0.76 (0.74–0.79)	0.84 (0.82–0.87)	0.88 (0.87–0.89)
SVM	0.71 (0.64–0.78))	0.90 (0.78–1.00)	0.62 (0.51–0.73)	0.49 (0.37–0.62)	0.94 (0.89–1.00)	0.86 (0.78–0.93)
OA v.s. RA						
DT	0.78 (0.73–0.83)	0.90 (0.85–0.95)	0.77 (0.70–0.84)	0.68 (0.58–0.78)	0.89 (0.82–0.96)	0.79 (0.74–0.83)
RF	0.80 (0.75–0.85)	0.84 (0.78–0.90)	0.84 (0.80–0.88)	0.86 (0.79–0.92)	0.68 (0.52–0.85)	0.87 (0.82–0.92)
SVM	0.66 (0.56–0.70)	0.66 (0.56–0.70)	0.79 (0.72–0.82	0.47 (0.33–0.60)	0.93 (0.88–0.99)	0.82 (0.80–0.85)

*DT, decision trees; RF, random forests; SVM, support vector machines

**Table 7:** Improved predictive performance using only selected features in decision trees, random forests, and support vector machines for healthy controls (HCs) versus rheumatoid arthritis (RA) patients and osteoarthritis (OA) versus RA patients

Algorithm*	Accuracy	Precision	F1 score	Sensitivity	Specificity	AUC	Selected feature
HC v.s. RA							
DT	0.84 (0.80–0.87)	0.92 (0.88–0.95)	0.80 (0.76–0.85)	0.73 (0.67–0.80)	0.93 (0.89–0.97)	0.86 (0.82–0.89)	IgM anti-HPT^78−108^ HNE IgG anti-THRB^328−345^
RF	0.87 (0.85–0.90)	0.91 (0.87–0.95)	0.85 (0.82–0.88)	0.81 (0.76–0.87)	0.93 (0.91–0.96)	0.92 (0.90–0.95)	IgM anti-HPT^78−108^ HNE IgM anti-HPT^78−108^ IgG anti-HPT^78−108^ IgG anti-THRB^328−345^ HNE adducts
SVM	0.71 (0.62–0.80)	0.97 (0.91–1.00)	0.56 (0.42–0.70)	0.42 (0.27–0.58)	0.98 (0.95–1.00)	0.82 (0.75–0.89)	IgM anti-HPT^78−108^ HNE IgG anti-HPT^78−108^ HNE IgM anti IGKC^2−19^ IgM anti-CFAH^1121−1230^ HNE
OA v.s. RA							
DT	0.81 (0.75–0.87)	0.94 (0.89–0.98)	0.82 (0.76–0.89)	0.74 (0.62–0.83)	0.93 (0.88–0.98)	0.84 (0.79–0.89)	IgM anti-HPT^78−108^ HNE IgG anti-THRB^328−345^ IgG anti-IGKC^2−19^
RF	0.85 (0.81–0.89)	0.87 (0.82–0.91)	0.87 (0.82–0.92)	0.88 (0.79–0.97)	0.79 (0.71–0.87)	0.92 (0.88–0.96)	IgM anti-HPT^78−108^ HNE IgG anti-HPT^78−108^ HNE IgG anti-IGKC^2−19^ IgM anti-CFAH^1121−1230^ HNE
SVM	0.68 (0.61–0.76)	0.68 (0.61–0.76)	0.81 (0.75–0.86)	0.53 (0.31–0.66)	0.94 (0.89–0.98)	0.88 (0.83–0.93)	IgM anti-HPT^78−108^ HNE IgG anti-THRB^328−345^

*DT, decision trees; RF, random forests; SVM, support vector machines

## Discussion

To the best of our knowledge, this is the first study to investigate autoantibodies isotypes against unmodified and HNE-modified peptides, its correlation with activity of disease in Taiwanese women with RA, and associations of risks for RA compared to HCs. However, a critical limitation should be noted due to our samples used in this study were not strictly selected during the disease progression of RA. Therefore, the efficacy of this test may be affected. Levels of HNE-protein adducts in RA patients were greater than the levels in HCs (Additional file 1: Table S1), which is consistent with results from a previous study [24]. Barrera et al*.* suggested that HNE-protein adducts also featured a pathogenic contribution of oxidative stress [43]. HNE-protein adducts, OSEs, are recognized as danger signals by innate immune receptors, such as the lectin-like oxidized LDL receptor 1 (LOX1) [44, 45]. HNE-protein adducts (OR 2.413) showed a risk for RA development (Table 4). Chronic inflammation can be triggered by the accumulation of OSEs, an important target of innate immunity, which increases the risk of developing chronic inflammation [5]. Binder et al. indicated that IgM isotypes against OSEs can enhance the clearance and neutralization of proinflammatory effect [5, 16, 17]. If OSEs cannot be efficiently cleared, OSEs would act as damage-associated molecular patterns (DAMPs) that trigger sterile inflammation [46]. Pattern recognition receptors (PRRs) can recognize DAMPs and activate the innate immune response to trigger sterile inflammation [47]. LOX1, a cellular PRR, can recognize and bind to HNE to mediate its uptake and inflammatory effect in atherosclerosis [48]. Macrophages can take up the IgM-NAA-HNE complex by C1q-calreticulin-CD91-dependent or mannose-binding lectin (MBL) and MBL receptor-dependent mechanisms in chronic inflammatory diseases and atherosclerosis [49]. Siloşi et al*.* reported that B-1 cells produce NAAs (IgM > IgG > IgA) and pathogenic autoantibodies (IgG > IgM > IgA) [50]. However, the boundary line between natural immunity and pathogenic autoimmunity is unclear [50]. Further, IgM-NAAs can control IgG-NAA activity and regulate expression of natural IgG autoreactive repertoire by F(ab')2 fragments of IgG-NAAs in human and mice serum [51, 52]. Chen et al*.* proved that inhibition of Toll-like receptor (TLR) and IgG-immune complex-mediated inflammatory responses mediate anti-inflammatory features of IgM-NAAs [53]. Moreover, IgG-NAAs may be involved in autoimmune disease pathogenesis, including SLE, Sjögren’s syndrome, and Graves’ disease [50, 54], and anti-OSE NAAs themselves may have a protective effect. Further, we deduced that elevated levels of anti-OSE NAAs may be a risk index of RA development based on protein function and disease activity when oxidative stress occurs over a sustained period in patients with RA (Additional file 1: Table S1, Table 4). In this study, four different novel HNE-modified peptide adducts were identified: CFAH^1211−1230^, HPT^78−108^, IGKC^2−19^, and THRB^328−345^ (Table 1, Fig. 1).

The biological function of CFAH, a soluble inhibitor of the alternative complement pathway, is to inhibit the inflammatory response through oxidative stress and to protect host tissues from complement-mediated damage [55–57]. Okroj et al*.* reported that complement activation contributes to the pathological process of RA [58]. The complement system is a central innate immune system that participates in eliminating pathogens and promotes inflammatory responses [55]. Weismann et al*.* indicated that CFAH is able to bind MDA, and as an MDA-binding protein, it blocks the proinflammatory effects that induced by MDA in vivo in mice [59]. Moreover, it was reported that the HNE modification was unbound by CFAH [59]. However, we found that CFAH was modified with HNE at K1230 in RA patients (Table 1, Additional file 2: Figure S1C). Trojnár et al*.* identified three linear epitopes on serum CFAH (CFAH^1157−1171^, CFAH^1177−1191^, and CFAH^1207−1226^) in atypical hemolytic uremic syndrome (aHUS) [60]. Interestingly, CFAH^1211−1230^ is also an autoantigen in RA (Fig. 3a, c). The HNE-modified CFAH^1211−1230^ peptide can enhance autoantibody levels in patients with RA compared to patients with OA and HCs (Fig. 3b, d). Several studies demonstrated that aHUS was associated with the presence of autoantibodies against CFAH [56, 57]. Autoantibodies against CFAH are also present in significant ratios in RA [57]. Insufficient inhibition of CFAH activity may be caused by pathology- associated autoantibodies [56, 58]. Thus, high autoantibody titers against CFAH are not specific to RA, but may be important for pathologic processes in RA. However, IgG anti-CFAH^1121−1230^, IgG anti-CFAH^1121−1230^ HNE, IgM anti-CFAH^1121−1230^, and IgM anti-CFAH^1121−1230^ HNE were not significantly correlated with DAS28-CRP, RF, CRP, ESR, or HNE-protein adducts, respectively (Table 3); but, IgG anti-CFAH^1121−1230^ (OR 3.293), IgG anti-CFAH^1121−1230^ HNE (OR 2.808), IgM anti-CFAH^1121−1230^ (OR 5.204), and IgM anti-CFAH^1121−1230^ HNE (OR 2.700) showed risks for RA development (Table 4).

HPT is a hemoglobin-binding protein that can prevent oxidative damage to organs and participates in activating innate and adaptive immune responses [61]. Increased synovial fluid (SF) and serum HPT levels found in RA patients were associated with inflammation and tissue destruction [62]. Yildirim et al*.* indicated that serum HPT was an acute-phase protein and significantly correlated with disease activity in patients with RA [63]. In this study, we identified one novel HNE modification at C92 on HPT^78–108^ in RA patients (Table 1, Additional file 2: Figure S1D). Korngold indicated that the HPT-anti-HPT reaction can block HPT-hemoglobin-binding action [64]. Muta et al*.* reported that the level of the anti-HPT antibody in serum increased and the level of HPT decreased after febrile non-hemolytic transfusion reactions (FNHTRs) [3]. In this study, higher levels of HPT in serum were greatly 1.24-fold higher (*p* = 0.041) in RA patients than in HCs (Fig. 2b). IgG and IgM against HPT^78−108^ and HPT^78−108^ HNE were greatly higher in RA patients than in HCs (Fig. 3e–h). Thus, we inferred that high levels of autoantibodies against HPT may inhibit HPT's function and play a role in the risk of developing RA. Interestingly, IgM anti-HPT^78−108^ HNE was significantly positively correlated with DAS28-CRP (*r* = 0.2703) and RF (*r* = 0.614), and IgG anti-HPT^78−108^ HNE was greatly positively correlated with anti-CCP (*r* = 0.2782), but IgG anti-HPT^78−108^, IgG anti-HPT^78−108^ HNE, and IgM anti-HPT^78−108^ were not significantly correlated with DAS28-CRP, RF, anti-CCP, CRP, ESR, or HNE-protein adducts (Table 3). Further, IgM anti-HPT^78−108^ (OR 2.695) and IgM anti-HPT^78−108^ HNE (OR 5.235) exhibited a risk of RA development (Table 4, Additional file 5: Table S2).

RFs are autoantibodies against the fragment crystallizable (Fc) region of IgG that, via antigenic stimulation, acts against an abnormal immune response from the host’s natural antibody repertoire [65]. The IgM RF is commonly measured in clinical practice and serves as a marker of RA, other rheumatic diseases, and chronic infections [66]. Sidorov et al*.* found that the human regulatory RF (regRF) can be induced by the hinge region of Fc fragments of homologous IgG and can prevent rheumatic diseases [2]. RF production can also be stimulated by modified IgG, including agalactosyl IgG, or advanced glycated end-product (AGE)-damaged IgG that are associated with more-severe RA and can play a meaningful role in pathogenesis of RA [67, 68]. In this study, we identified two novel HNE modifications at A4 and A5 on IGKC^2−19^, which is located on the IgG light chain in RA patients (Table 1, Additional file 2: Figure S1E). IgG and IgM against IGKC^2−19^ and IGKC^2−19^ HNE were greatly higher in patients with RA than in HCs (Fig. 3i–l). Thus, we inferred that high levels of autoantibodies against IGKC may promote the risk of developing RA. Indeed, IgM anti-IGKC^2−19^ was greatly positively correlated with DAS28-CRP (*r* = 0.2816), RF (*r* = 0.5674), ESR (*r* = 0.2692), and HNE-protein adducts (*r* = 0.2667). However, IgM anti-IGKC^2−19^ HNE was significantly correlated with RF (*r* = 0.5404), ESR (*r* = 0.2985), and HNE-protein adducts (*r* = 0.2709) (Table 3). Interestingly, IgG anti-IGKC^2−19^ (*r* = − 0.3538) and IgG anti-IGKC^2−19^ HNE (*r* = − 0.3432) were significantly negatively correlated with DAS28-CRP (Table 3). Additional research is required to determine whether both IgG-NAAs and IgG anti-HNE NAAs have functions similar to regRF. Further, IgG anti-IGKC^2−19^ (OR 4.679), IgG anti-IGKC^2−19^ HNE (OR 3.206), IgM anti-IGKC^2−19^ (OR 12.665), and IgM anti-IGKC^2−19^ HNE (OR 8.095) showed risks for RA development (Table 4).

RA is characterized by activation of both inflammatory and coagulation processes resulting in erosion of the joints [69]. THRB is transformed into thrombin by a prothrombinase when injury occurs to tissues and then changes via fibrinogen to form fibrin in a coagulation process [70]. Ohba et al*.* suggested that high levels of thrombin activity in SF via strong mitogenic activity toward synovial fibroblast-like cells play a significant role in the RA pathogenesis [71]. Yang et al*.* reported that anti-THRB autoantibodies can display prothrombinase activity and contribute to thrombosis in anti-phospholipid syndrome (APS) and SLE [72]. In this study, novel HNE modifications at K344 on THRB ^328–345^ in RA patients were identified (Table 1, Additional file 2: Figure S1F). Next, levels of IgG and IgM against THRB^328−345^ and THRB^328−345^ HNE were greatly higher in patients with RA than in HCs (Fig. 3m–p). Thus, high levels of anti-THRB autoantibodies may be considered as a risk factor for RA. Interestingly, IgG anti-THRB^328−345^ was greatly positively correlated with DAS28-CRP (*r* = 0.2703) and RF (*r* = 0.6140), IgG anti-THRB^328−345^ HNE was significantly positively correlated with RF (*r* = 0.3072) and anti-CCP (*r* = 0.2549), IgM anti-THRB^328−345^ HNE was greatly positively correlated with RF (*r* = 0.2845) and ESR (*r* = 0.2597), but IgM anti-THRB^328−345^ was significantly negatively correlated with the HNE-protein adduct (*r* = − 0.2796) as shown in Table 3. Further, IgG anti-THRB^328−345^ (OR 5.761), IgG anti-THRB^328−345^ HNE (OR 9.542), and IgM anti-THRB^328−345^ HNE (OR 5.043) exhibited risks for RA development (Table 4).

Several previous studies showed the feasibility in early diagnosis of autoimmune diseases using a machine learning application for RA. Rodrigo Torres et al*.* suggested that feature selection can be a powerful tool in biomarker discovery [73]. Therefore, we believe that with appropriate proteomic data and machine learning algorithms, the biomarker candidates we developed can be optimized into a set of highly accurate features. In our experiment, we incorporated feature ranking and a forward selection method to identify IgM anti-HPT^78−108^ and HNE-protein adducts that can identify RA from HC combined with random forest algorithm. We then compared our results with the accuracy of anti-CCP which was considered a well performance biomarker in RA. Other studies reported that the sensitivity and specificity of anti-CCP were 63% ~ 91.4% and 69.7% ~ 97.6%, respectively [74–77]. However, we found only 50% of positive anti-CCP in RA samples. Moreover, consistency was observed between statistical analyses of odds ratio and prediction results of classification. Our results from statistical models, feature selection, and machine learning classifiers supported that IgM anti-HPT^78−108^ HNE, IgM anti-IGKC^2−19^, and IgM anti-IGKC^2−19^ HNE showed potential to be developed as biomarkers for RA.

## Conclusions

In the present study, we found that some IgG- and IgM-NAAs and anti-HNE IgM-NAAs may be correlated with activity of disease and inflammation in RA. We concluded that increased levels of IgM anti-HPT^78−108^ HNE, IgM anti-IGKC^2−19^, and IgM anti-IGKC^2−19^ HNE in serum can be used as diagnostic biomarkers of RA, and high levels of IgM anti-HPT^78−108^ HNE, IgM anti-IGKC^2−19^, and IgG anti-THRB^328−345^ are related to increased disease activity during RA development.

## Supplementary Information

**Additional file 1: Table S1.** Demographic and clinical characteristics of individual subjects contributing to serum for healthy controls (HCs), and patients with osteoarthritis (OA) and rheumatoid arthritis (RA).

**Additional file 2: Fig. S1.** Differential 4-hydroxy-2-nonenal (HNE)-modified peptide adducts were re-analyzed through PEAKS 7 using previous MS/MS spectra (ProteomeXchange: PXD004546). Acquired MS/MS spectra were obtained through pooled concanavalin (Con) A-captured serum proteins (nine rheumatoid arthritis (RA) and nine healthy control (HC) pooled samples), 1-D SDS-PAGE, in-gel digestion, and nano-LC-MS/MS (A). HNE reacts with amino acid residues of proteins to form HNE-protein adducts by Michael addition and Schiff base adducts, respectively (B). Representative MS/MS spectrum of the 1211-SHTLRTTCWDGKLEYPTCAK-1230 peptide sequence and the modified peptide bearing an HNE modification at the K1230 residue in RA patients (C). A representative MS/MS spectrum of the peptide sequence 78-AVGDKLPECEADDGCPKPPEIAHGYVEH SVR-108 and the modified peptide bearing the HNE modification at the C92 residue in RA patients (D). The MS/MS spectrum 2-TVAAPSVFIFPPSDEQLK-19 and the modified peptide bearing the HNE modification at the A5 residue in RA patients (E, upper panel); 2-TVAAPSVFIFPPSDEQLK-19 and the modified peptide bearing the HNE modification at the A4 residues in RA patients and HCs (E, bottom panel). Representative MS/MS spectrum of 328-TFGSGEADCGLRPLFEKK-345 and the modified peptide bearing the HNE modification at the K344 residue in RA (F). The MS/MS spectrum 284-HRTGDEITYQCRNGFYPATRGNTAK-308 and the modified peptide bearing the HNE modification at the K308 residue in HCs (G). A representative MS/MS spectrum of the peptide sequence 162-ILGGHLDAK-170 and the modified peptide bearing the HNE modification at the A169 residues in HCs (H). Representative MS/MS spectrum of 83-VYACEVTHQGLSSPVTKSFNR-103 and the modified peptide bearing the HNE modification at the Q91 residue in HCs (I). The MS/MS spectrum 328-TFGSGEADCGLRPLFEK-344 and the modified peptide bearing the HNE modification at the C336 and L341 residues in HCs (J).

**Additional file 3.** Supplementary methods.

**Additional file 4: Fig. S2.** Comparison of receiver operating characteristics (ROC) curves from unselected features and selected features in (A) decision tree, (B) random forest classifier, and (C) support vector machine classifier.

**Additional file 5: Table S2.** Sequences of unmodified and 4-hydroxy-2-nonenal (HNE)-modified peptides.

## Data Availability

The data that support the findings of this study are available from Shuang-Ho Hospital (New Taipei City, Taiwan) but restrictions apply to the availability of these data, which were used under license for the current study, and so are not publicly available. Data are however available from the authors upon reasonable request and with permission of the hospital.

## Abbreviations

1-D SDS-PAGE: One-dimensional sodium dodecylsulfate polyacrylamide gel electrophoresis
ACR: American College of Rheumatology
AD: Alzheimer's disease
aHUS: Atypical hemolytic uremic syndrome
ALD: Alcoholic liver disease
AUC: Area under the receiver operating characteristic curve
CBB: Coomassie brilliant blue
CD: Cardiovascular disease
CFAH: Complement factor H
Con A: Concanavalin A
COPD: Chronic obstructive pulmonary disease
CRP: C-reactive protein
DAMP: Damage-associated molecular pattern
DAS28-CRP: Disease activity score in 28 joints
DM: Diabetes mellitus
DMARD: Disease-modifying anti-rheumatic drug
ELISA: Enzyme-linked immunosorbent assay
ESR: Erythrocyte sedimentation rate
FDR: False detection rate
HC: Healthy control
HNE: 4-Hydroxy-2-nonenal
Ig: Immunoglobulin
HPT: Haptoglobin
IGKC: Immunoglobulin kappa chain C region
IP: Immunoprecipitation
MBL: Mannose-binding lectin
MCI: Mild cognitive impairment
MKHD: Menkis kinky hair disease
MDA: Malondialdehyde
NAA: Natural autoantibody
nano-LC–MS/MS: Nano-liquid chromatography-tandem mass spectrometry
NSAID: Non-steroidal anti-inflammatory drug
OA: Osteoarthritis
ANOVA: Analysis of variance
OR: Odds ratio
CI: Confidence interval
OSE: Oxidation-specific epitope
PRR: Pattern recognition receptor
PTM: Post-translational modification
RF: Rheumatoid factor
Anti-CCP: Anti-cyclic citrullinated peptide
ROC: Receiver operating characteristic
SF: Synovial fluid
SLE: System lupus erythematosus
THRB: Prothrombin
