# Supplementary material for: Isotypes of autoantibodies against novel differential 4-hydroxy-2-nonenal-modified peptide adducts in serum is associated with rheumatoid arthritis in Taiwanese women
Source: BMC Med Inform Decis Mak. 2021 Feb 10;21:49. doi: 10.1186/s12911-020-01380-y (PMC7874460; doi:10.1186/s12911-020-01380-y)
Supplement: Supplementary file 1 — Additional file 1: Table S1. Demographic and clinical characteristics of individual subjects contributing to serum for healthy controls (HCs), and patients with osteoarthritis (OA) and rheumatoid arthritis (RA). [file 12911_2020_1380_MOESM1_ESM.docx]

|  | Supplementary Table 1. | | | |
| --- | --- | --- | --- | --- |
| Characteristic | | **HC ^a^** | **OA ^b^** | **RA ^b^** |
|  |  | n = 60 | n = 35 | n = 60 |
| **Age (years)** | | 54.3 ± 8.70 | 56.2 ± 11.44 | 54.8 ± 10.47 |
| **Gender** | |  |  |  |
| Female | | 60 | 35 | 60 |
| **Disease duration (median in years)** | | N.A. **^c^** | N.A. | 5.4 ± 6.41 |
| **DAS 28-CRP** | | N.A. | N.A. | 4.4 ± 1.67 |
| **Clinical tests** | |  |  |  |
| RF - positive (%) | | 0 | 0 | 75.9 |
| Anti-CCP - positive (%) | | 0 | N.A. | 55.0 |
| CRP - positive (%) | | N.A. | 20 | 26.7 |
| ESR - positive (%) | | N.A. | 65.7 | 81.7 |
| **Current therapy** | |  |  |  |
| NSAIDs (%) | | N.A. | 65 | 42.9 |
| DMARDs (%) | | N.A. | 11.4 | 93.3 |
| Other (e.g. steroids) (%) | | N.A. | 55 | 31.4 |
| **HNE-protein adduct (µg/mL) ^d^** | | 2.73 ± 1.252 | 2.81 ± 1.195 | 3.26 ± 0.801 |
| a. HCs were tested by Union Clinical Laboratory in Taiwan: Rheumatoid factor (Latex RF reagent, Siemens Medical Solutions Diagnostics, USA; cutoff of < 15.0 IU/mL); INOVA QUANTA Lite CCP3 IgG ELISA (INOVA DIAGNOSTICS, INC; cutoff of < 20 U).  b. Patient samples were analyzed at the Department of Laboratory Medicine, Shuang Ho Hospital, Taipei Medical University, Taiwan: Rheumatoid factor (Latex RF reagent, Siemens Medical Solutions Diagnostics; cutoff of < 15.0 IU/mL), “DIESSE” CHORUS ANTI-CCP (DIESSE DIAGNOSTICA SENESE S.P.A; cutoff of <.7.0 AU/mL); CRP (CardioPhase® hsCRP, SIEMENS, Munich, Germany; cutoff of < 0.5 mg/dL) and ESR 1h (VACUETTE®, Greiner Bio-One, Kremsmünster, Austria; cutoff of < 10 mm/1 hr).  c. N.A., Not available.  d. RA vs. HC, *p* = 0.0062 ; RA vs. OA, *p* = 0.0311. | | | | |
