# Supplementary material for: Isotypes of autoantibodies against novel differential 4-hydroxy-2-nonenal-modified peptide adducts in serum is associated with rheumatoid arthritis in Taiwanese women
Source: BMC Med Inform Decis Mak. 2021 Feb 10;21:49. doi: 10.1186/s12911-020-01380-y (PMC7874460; doi:10.1186/s12911-020-01380-y)
Supplement: Supplementary file 2 — Additional file 2: Fig. S1. Differential 4-hydroxy-2-nonenal (HNE)-modified peptide adducts were re-analyzed through PEAKS 7 using previous MS/MS spectra (ProteomeXchange: PXD004546). Acquired MS/MS spectra were obtained through pooled concanavalin (Con) A-captured serum proteins (nine rheumatoid arthritis (RA) and nine healthy control (HC) pooled samples), 1-D SDS-PAGE, in-gel digestion, and nano-LC-MS/MS (A). HNE reacts with amino acid residues of proteins to form HNE-protein adducts by Michael addition and Schiff base adducts, respectively (B). Representative MS/MS spectrum of the 1211-SHTLRTTCWDGKLEYPTCAK-1230 peptide sequence and the modified peptide bearing an HNE modification at the K1230 residue in RA patients (C). A representative MS/MS spectrum of the peptide sequence 78-AVGDKLPECEADDGCPKPPEIAHGYVEH SVR-108 and the modified peptide bearing the HNE modification at the C92 residue in RA patients (D). The MS/MS spectrum 2-TVAAPSVFIFPPSDEQLK-19 and the modified peptide bearing the HNE modification at the A5 residue in RA patients (E, upper panel); 2-TVAAPSVFIFPPSDEQLK-19 and the modified peptide bearing the HNE modification at the A4 residues in RA patients and HCs (E, bottom panel). Representative MS/MS spectrum of 328-TFGSGEADCGLRPLFEKK-345 and the modified peptide bearing the HNE modification at the K344 residue in RA (F). The MS/MS spectrum 284-HRTGDEITYQCRNGFYPATRGNTAK-308 and the modified peptide bearing the HNE modification at the K308 residue in HCs (G). A representative MS/MS spectrum of the peptide sequence 162-ILGGHLDAK-170 and the modified peptide bearing the HNE modification at the A169 residues in HCs (H). Representative MS/MS spectrum of 83-VYACEVTHQGLSSPVTKSFNR-103 and the modified peptide bearing the HNE modification at the Q91 residue in HCs (I). The MS/MS spectrum 328-TFGSGEADCGLRPLFEK-344 and the modified peptide bearing the HNE modification at the C336 and L341 residues in HCs (J). [file 12911_2020_1380_MOESM2_ESM.pptx]

## Slide 1
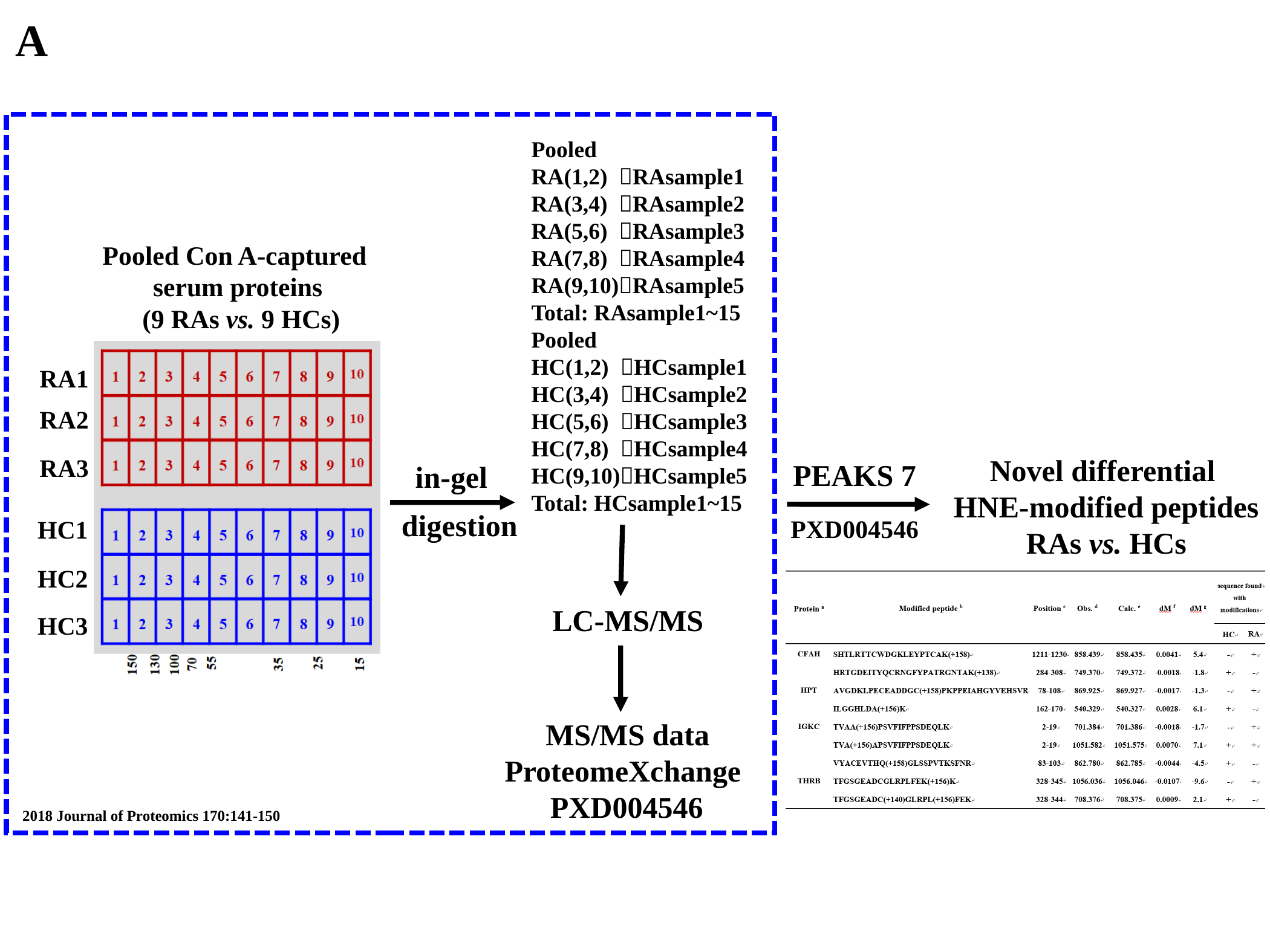

A
Pooled
RA(1,2) RAsample1
RA(3,4) RAsample2
RA(5,6) RAsample3
RA(7,8) RAsample4
RA(9,10)RAsample5
Total: RAsample1~15
Pooled
HC(1,2) HCsample1
HC(3,4) HCsample2
HC(5,6) HCsample3
HC(7,8) HCsample4
HC(9,10)HCsample5
Total: HCsample1~15
Pooled Con A-captured
serum proteins
 (9 RAs vs. 9 HCs)
RA1
RA2
Novel differential
HNE-modified peptides
RAs vs. HCs
RA3
PEAKS 7
in-gel
digestion
PXD004546
HC1
HC2
LC-MS/MS
HC3
MS/MS data
ProteomeXchange
PXD004546
2018 Journal of Proteomics 170:141-150

## Slide 2
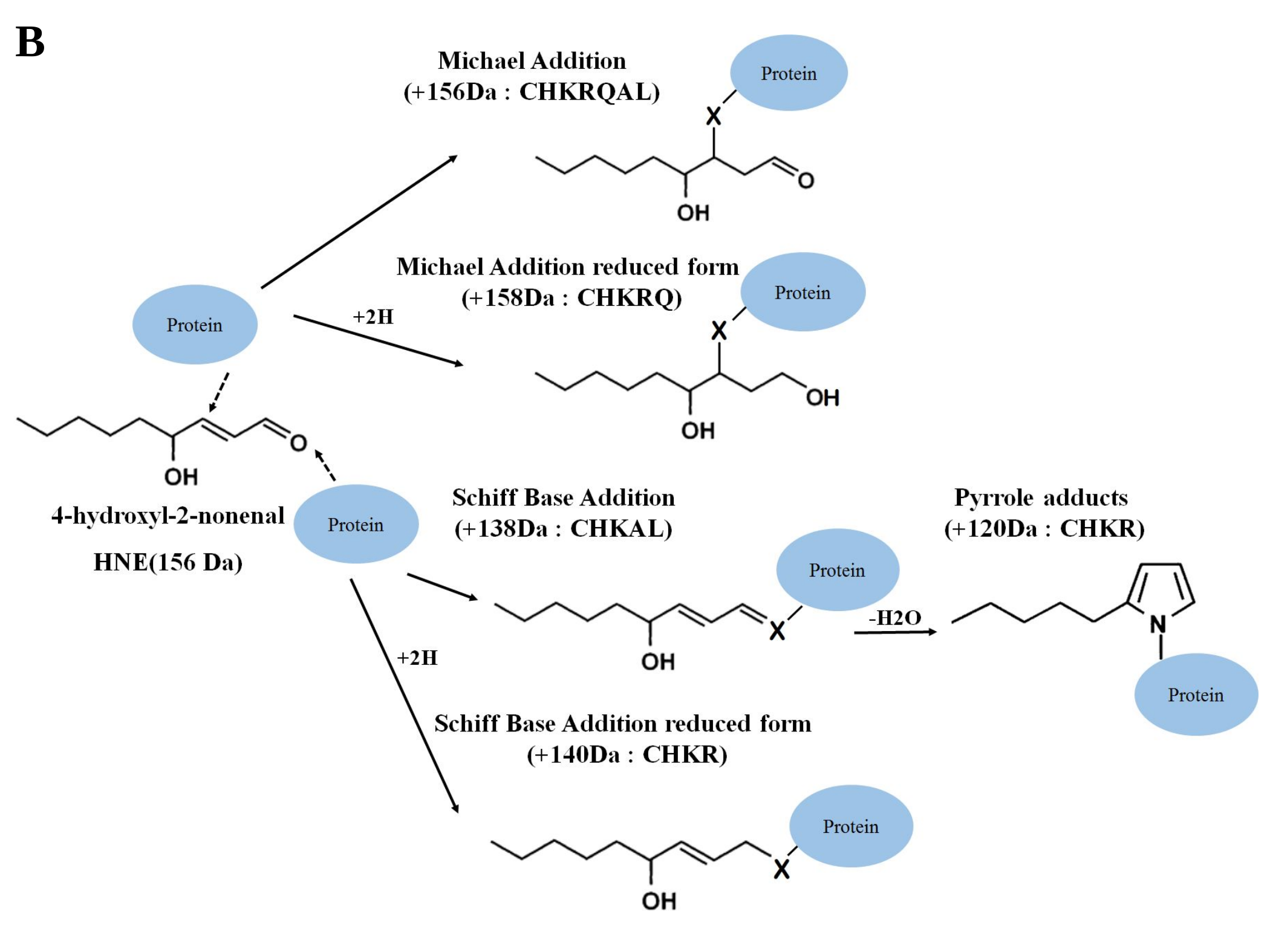

B

## Slide 3
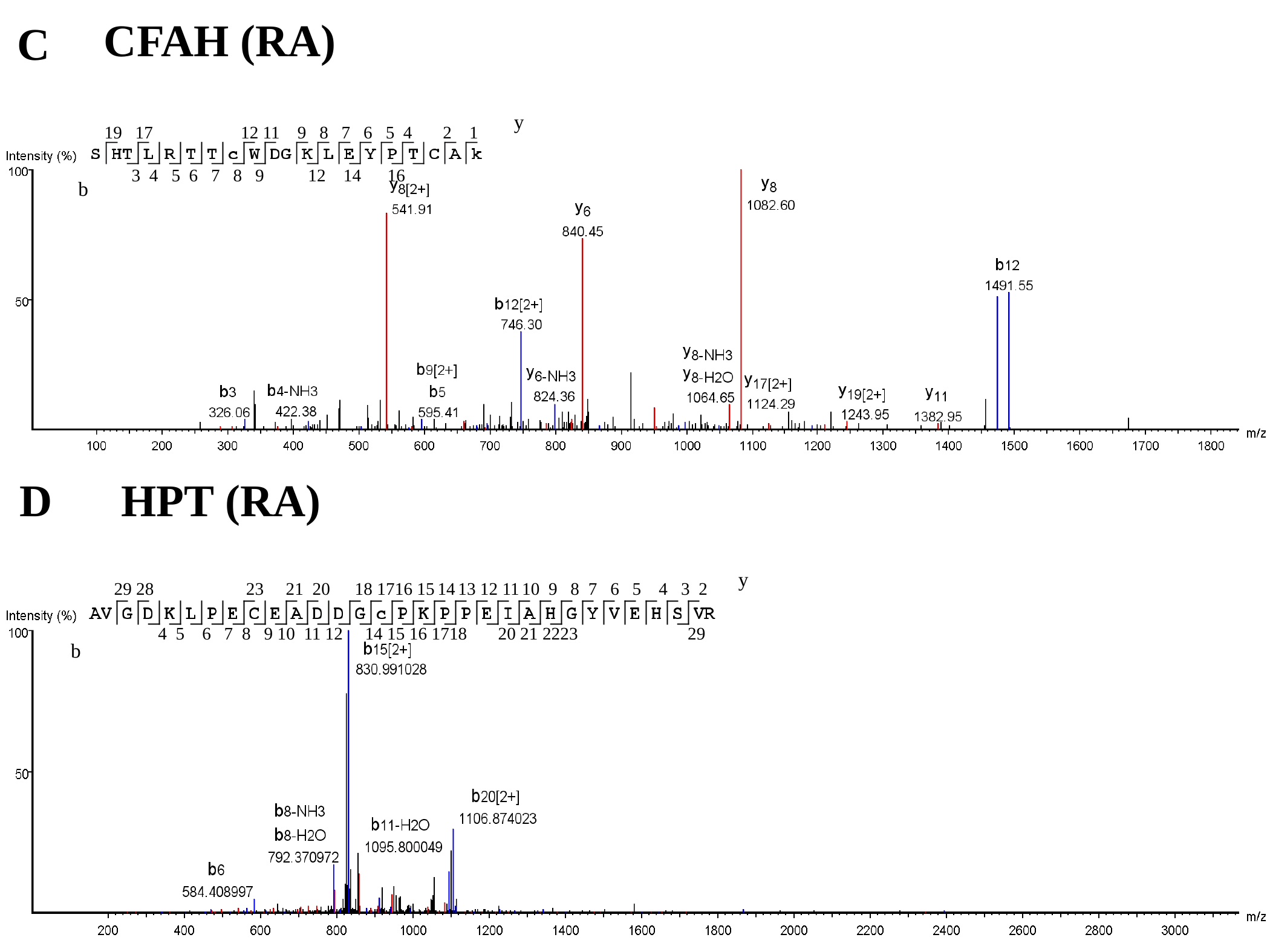

CFAH (RA)
C
y
 19 17 12 11 9 8 7 6 5 4 2 1
3 4 5 6 7 8 9 12 14 16
b
HPT (RA)
D
y
 29 28 23 21 20 18 1716 15 14 13 12 11 10 9 8 7 6 5 4 3 2
4 5 6 7 8 9 10 11 12 14 15 16 1718 20 21 2223 29
b

## Slide 4
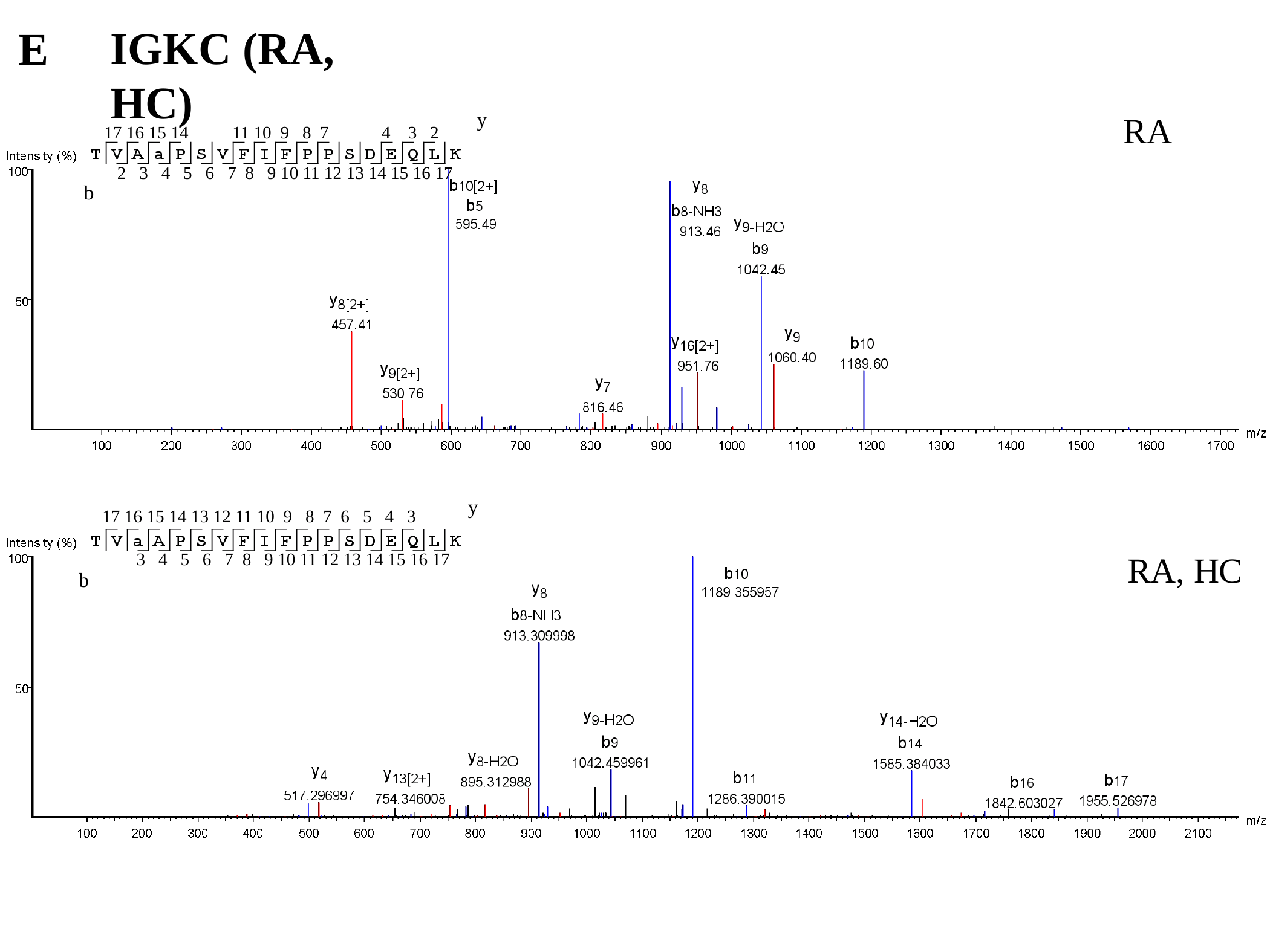

IGKC (RA, HC)
E
y
RA
 17 16 15 14 11 10 9 8 7 4 3 2
2 3 4 5 6 7 8 9 10 11 12 13 14 15 16 17
b
y
17 16 15 14 13 12 11 10 9 8 7 6 5 4 3
 3 4 5 6 7 8 9 10 11 12 13 14 15 16 17
RA, HC
b

## Slide 5
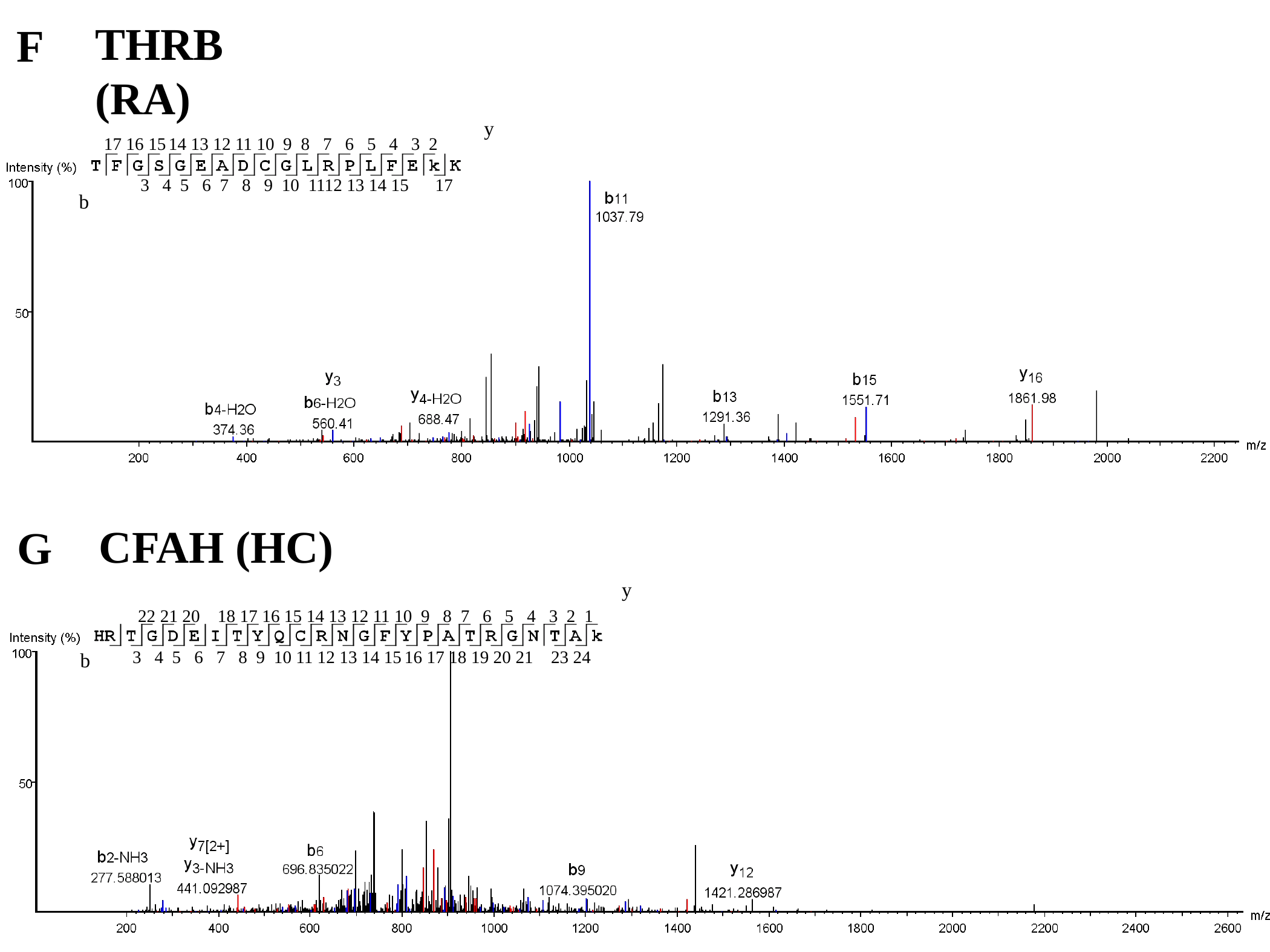

THRB (RA)
F
y
 17 16 15 14 13 12 11 10 9 8 7 6 5 4 3 2
3 4 5 6 7 8 9 10 1112 13 14 15 17
b
CFAH (HC)
G
y
22 21 20 18 17 16 15 14 13 12 11 10 9 8 7 6 5 4 3 2 1
3 4 5 6 7 8 9 10 11 12 13 14 15 16 17 18 19 20 21 23 24
b

## Slide 6
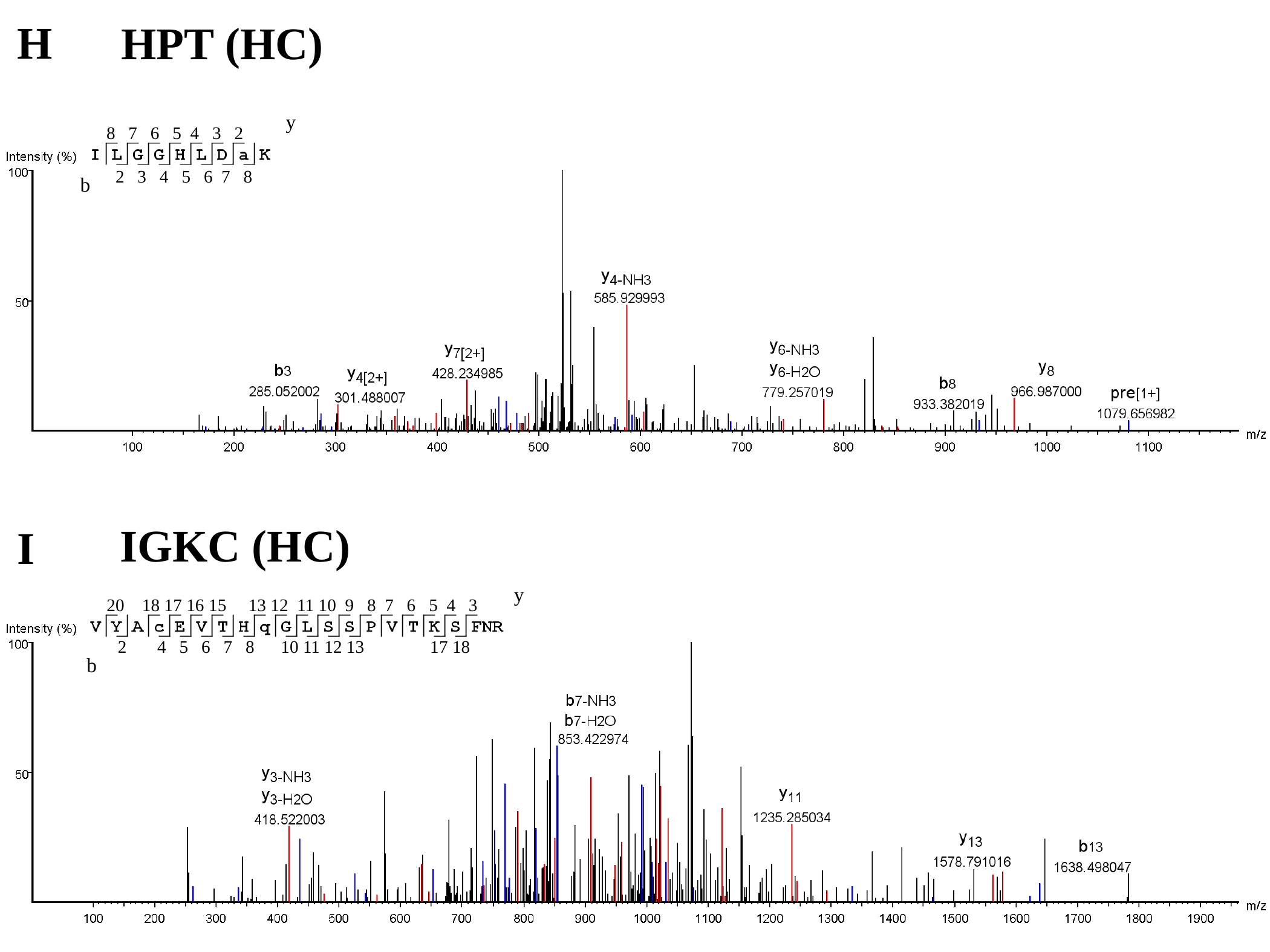

H
HPT (HC)
y
8 7 6 5 4 3 2
2 3 4 5 6 7 8
b
IGKC (HC)
I
y
 20 18 17 16 15 13 12 11 10 9 8 7 6 5 4 3
2 4 5 6 7 8 10 11 12 13 17 18
b

## Slide 7
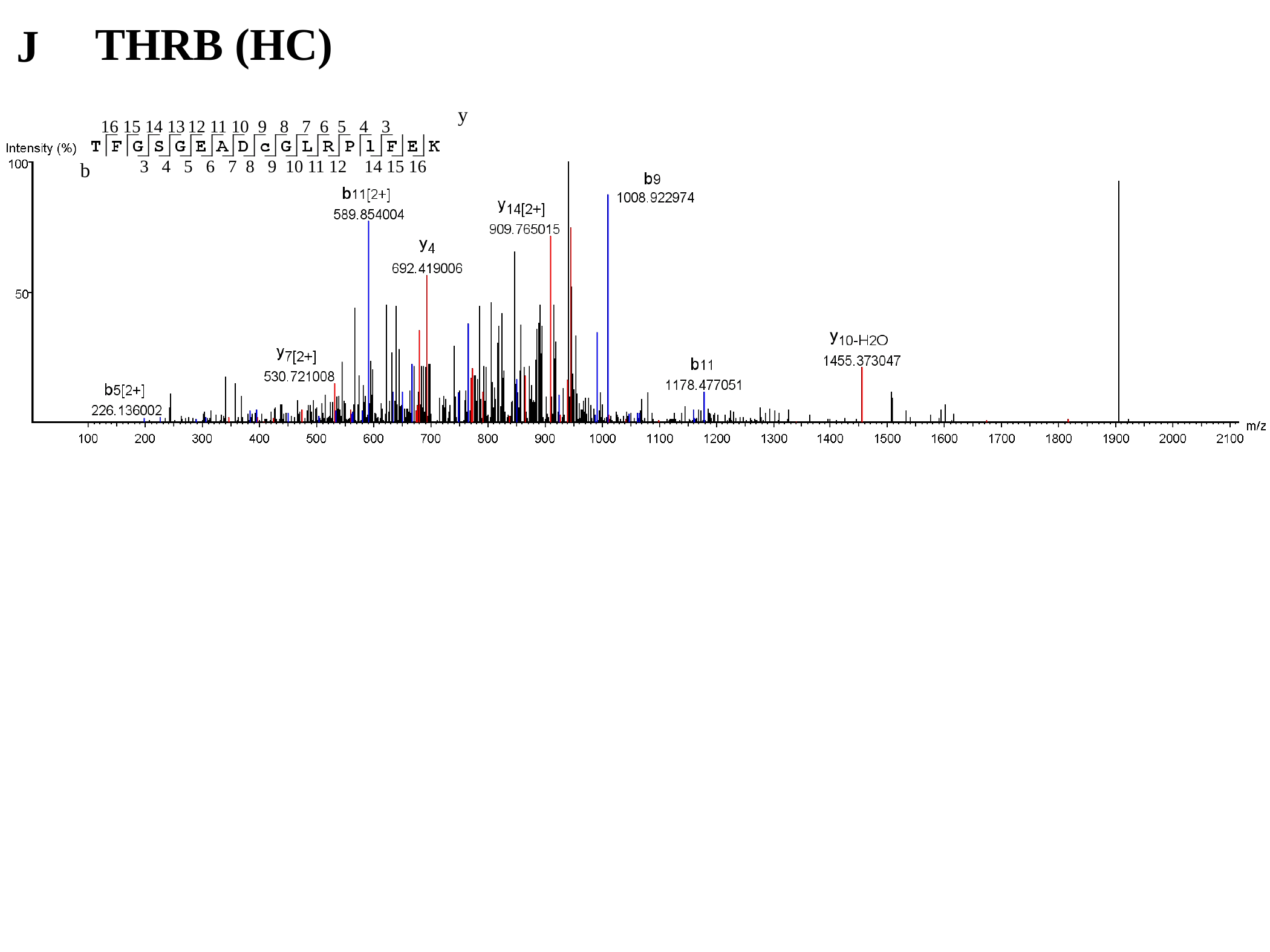

THRB (HC)
J
y
16 15 14 13 12 11 10 9 8 7 6 5 4 3
3 4 5 6 7 8 9 10 11 12 14 15 16
b
