# Supplementary material for: Isotypes of autoantibodies against novel differential 4-hydroxy-2-nonenal-modified peptide adducts in serum is associated with rheumatoid arthritis in Taiwanese women
Source: BMC Med Inform Decis Mak. 2021 Feb 10;21:49. doi: 10.1186/s12911-020-01380-y (PMC7874460; doi:10.1186/s12911-020-01380-y)
Supplement: Supplementary file 3 — Additional file 3. Supplementary methods. [file 12911_2020_1380_MOESM3_ESM.docx]

**Supplementary information**

1. **Supplementary methods**

**1.1 Concanavalin (Con) A affinity chromatography**

Pooled serum (100 µL) was mixed with agarose-bound Con A (300 μL; Vector Laboratories, Burlingame, CA, USA) at 4 °C and kept overnight. Con A-bound serum was captured by washing Con A with an 8× binding buffer (1 mM MnCl_2_, 1 mM CaCl_2_, 25 mM Tris, and 500 mM NaCl; pH 7.6), eluted six times with 200 μL of elution buffer (500 mM α-methyl mannoside in 20 mM Tris, pH 7.6), and enriched Con A-bound proteins by Microcon YM-10 (Millipore, Billerica, MA, USA). Con A-captured serum proteins were quantified using a Pierce™ Coomassie Plus (Bradford) Assay Kit (Thermo Scientific, Waltham, MA, USA).

**1.2 Parameters of PEAKS 7 software**

The maximum missed cleavage was set to 2. The mass tolerance of precursor ions was set to 20 ppm, and the fragment ion tolerance with a monoisotopic mass was set at 1.0 Da. Using a false detection rate (FDR) of 1.0% and a significant score (-10lgP) for peptides of ≥ 22, proteins with a target-decoy database search were accepted if they had at least two detected peptides and two unique peptides. Maximum variable post-translational modifications (PTMs) were set to 5.

**1.3 Determination of the serum protein concentration**

A Pierce™ Coomassie Plus (Bradford) Assay Kit (Thermo Scientific) was used to quantify protein concentrations. Briefly, 200 µL of working reagents were added to a flat-bottomed 96-well plate. Next, 4 μL of duplicate serum samples and bovine serum albumin (BSA) standards were loaded on the plate and incubated for 10 min at 37 °C. The measurement of absorbance was set at 595 nm. A standard curve of protein quantification was set up using the BSA standard concentration (0~1500 μg/mL).

**1.4 Immunoprecipitation (IP)**

4-Hydroxy-2-nonenal (HNE)-protein adducts were immunoprecipitated from pooled Con A-captured serum samples (32 healthy controls (HCs) and 32 rheumatoid arthritis (RA) patients). Percentages of SDS-PAGE (Hoefer^®^, Holliston, MA, USA) gel and IP loading amounts of pooled Con A-captured serum protein samples were 8% and 20 µg, 10% and 20 µg, 12% and 5 µg, and 8% and 20 µg for complement factor H (CFAH), haptoglobin (HPT), immunoglobulin kappa chain C region (IGKC), and prothrombin (THRB), respectively, and these were used in the IP-Western blot analysis. A mouse monoclonal anti-CFAH (MAB4779, R&D Systems, Minneapolis, MN, USA), mouse monoclonal anti-HPT (sc-69783, Santa Cruz Biotechnology, Dallas, TX, USA), rabbit polyclonal anti-IGKC (ab134083, Abcam, Cambridge, MA, USA), or rabbit polyclonal anti-THRB (H00002147-D01, Abnova, Taipei, Taiwan) antibody was coupled to 2 mg of Protein A Sepharose™ CL-4B (GE Healthcare Bio-Sciences, Pittsburgh, PA, USA) in 400 μL of NET-2 buffer (50 mM Tris-HCl, 150 mM NaCl, and 0.5% triton X100; at pH 7.4), then incubated with Con A-captured serum proteins, and mixed gently overnight at 4 °C. The beads were washed five times with 1 mL of NET-2* buffer (50 mM Tris-HCl at pH 7.4, 150 mM NaCl, and 0.05% triton X100) and centrifuged at 4000 rpm for 3 min. Finally, HNE modification of the immunoprecipitated protein was validated through sodium dodecylsulfate polyacrylamide gel electrophoresis (SDS-PAGE) and verified through Western blotting using a goat polyclonal anti-HNE antibody (MyBioSource, San Diego, CA, USA). Another duplicate protein gel stain used Coomassie brilliant blue (CBB) staining solution (Bio-Rad Laboratories) that was as a loading control.

**1.5 Western blotting**

SDS-PAGE was handled with the protocol of Laemmli [1]. The Western blotting used the percentages of SDS-PAGE gel and loaded amounts of serum proteins were 8% and 20 µg, 10% and 5 µg, 12% and 1 µg, and 8% and 20 µg for CFAH, HPT, IGKC, and THRB, respectively. Serum proteins were separated through an appropriate SDS-PAGE procedure (Hoefer^®^) in Tris-glycine buffer (25 mM Tris, 192 mM glycine, and 0.1% w/v SDS; pH 8.3) and transferred onto a polyvinylidene difluoride (PVDF) membrane in transfer buffer (25 mM Tris base, 192 mM glycine, and 20% methanol; pH 8.0; GE Healthcare Life Sciences, Piscataway, NJ, USA). Membrane was blocked with protein-free blocking buffer (BF01-1L, Visual Protein, Taipei, Taiwan) and followed incubation with antibodies including mouse monoclonal anti-CFAH (MAB4779, dilution 1:1000, R&D Systems), mouse monoclonal anti-HPT (sc-69783, dilution 1:20000, Santa Cruz Biotechnology), rabbit polyclonal anti- IGKC (ab134083, dilution 1:10000, Abcam), or rabbit polyclonal anti-THRB (H00002147-D01, dilution 1:5000, Abnova). The Western blotting used a goat polyclonal anti-HNE antibody (MBS536107, dilution 1:5000, MyBioSource) to detect HNE-modified proteins. After being washed, membrane was incubated with a horseradish peroxidase (HRP)-conjugated goat anti-rabbit (sc-2054) or anti-mouse (sc-2055) monoclonal IgG antibody (both dilution 1:10000; Santa Cruz Biotechnology) that was as the secondary antibody. The visualization of protein bands used the Luminata^TM^ Forte Western HRP Substrate (Millipore). The visualized band intensity was digitized and counted using the ImageQuant 400^TM^ Imager (GE Healthcare Life Sciences) and ImageJ software (National Institutes of Health, Bethesda, MD, USA). Western blotting was in duplicate. After Western blotting, the PVDF membranes stained with CBB (Bio-Rad Laboratories) that was as a loading control.

**1.6 Enzyme-linked immunosorbent assay (ELISA) for HNE-protein adduct quantification**

We adsorbed 100 µL of serum samples (10 μg/mL) or reduced/HNE-modified BSA (STA-335, Cell Biolabs) standards (0~10 μg/mL) on a flat-bottomed 96-well plate and followed incubation the plate at 37 °C for 2 h. After washing the plates with phosphate-buffered saline (PBS) containing 0.05% Tween 20 (PBST) and blocking them with protein-free blocking buffer (BF01-1L, Visual Protein, Taipei, Taiwan) at room temperature for 1 h, we added the HRP-conjugated goat anti-HNE antibody (MBS390040, dilution 1:5000, Cell Biolabs) and incubated samples and standards at 37 °C for 3 h. Subsequently, after washing the plates, we detected the bound antibody-HRP using the SureBlue Reserve^TM^ TMB Microwell Peroxidase Substrate (Kirkegaard & Perry Laboratories, Gaithersburg, MD, USA) after incubation for 30 min at room temperature. The color reaction was terminated by 1 N HCl, and the measurement of absorbance was set at 450 nm. Amounts of HNE-protein adducts in serum were measured in accordance to a standard HNE-modified BSA curve. Amounts of HNE-protein adducts are expressed as µg/mL.

**1.7 Detection of autoantibodies against unmodified and HNE-modified peptides**

Four peptides (1 mg/ml) were modified with 2 mM HNE in 0.1 M phosphate buffer (pH 7.4) at 37 °C for 3 h. The CFAH and HPT peptides were then subjected to reductive stabilization for 30 min at room temperature at a final concentration of 5 mM NaBH4 (stock concentration of 100 mM NaBH4 in 100 mM NaOH). HNE-modified peptide took a dialysis against PBS containing 1 mM EDTA changed with four times for 18 h at 4 °C. Diluted peptides (10 µg/mL) with PBS (pH 7.4) were absorbed onto a plate and then incubated overnight at 4 °C. After washing and blocking the plates, we incubated them with 100-fold diluted serum and PBS at 37 °C for 2 h. After washing the plates, we used rabbit anti-human IgG-HRP (dilution 1:10000, Santa Cruz Biotechnology) or rabbit anti-human IgM-HRP (dilution 1:10000, Santa Cruz Biotechnology) to detect the deposition of autoantibodies directed to a unmodified or HNE-modified peptide at 37 °C for 1 h. The antibody-HRP was reacted with the SureBlue Reserve^TM^ TMB Microwell Peroxidase Substrate (Kirkegaard & Perry Laboratories) and followed incubation for 10 min at room temperature; the color reaction was terminated with 1 N HCl, and the measurement of absorbance was set at 450 nm. All ELISA experiments were conducted by following ELISA Guidebook [2]. Three sample controls including HC, OA and RA with 2 replicates were placed in each plate to calculate CV% across wells and plates. The experiments were repeated if CV% were calculated over than 20.

[1] S. Cannon-Carlson, J. Tang, Modification of the Laemmli sodium dodecyl sulfate-polyacrylamide gel electrophoresis procedure to eliminate artifacts on reducing and nonreducing gels, Analytical biochemistry 246(1) (1997) 146-8.

[2] Crowther, J. R. (2000). ELISA Guidebook, The. doi:10.1385/1592590497
