# Supplementary figures and images for: Isotypes of autoantibodies against novel differential 4-hydroxy-2-nonenal-modified peptide adducts in serum is associated with rheumatoid arthritis in Taiwanese women
Source: BMC Med Inform Decis Mak. 2021 Feb 10;21:49. doi: 10.1186/s12911-020-01380-y (PMC7874460; doi:10.1186/s12911-020-01380-y)

## Slide 1
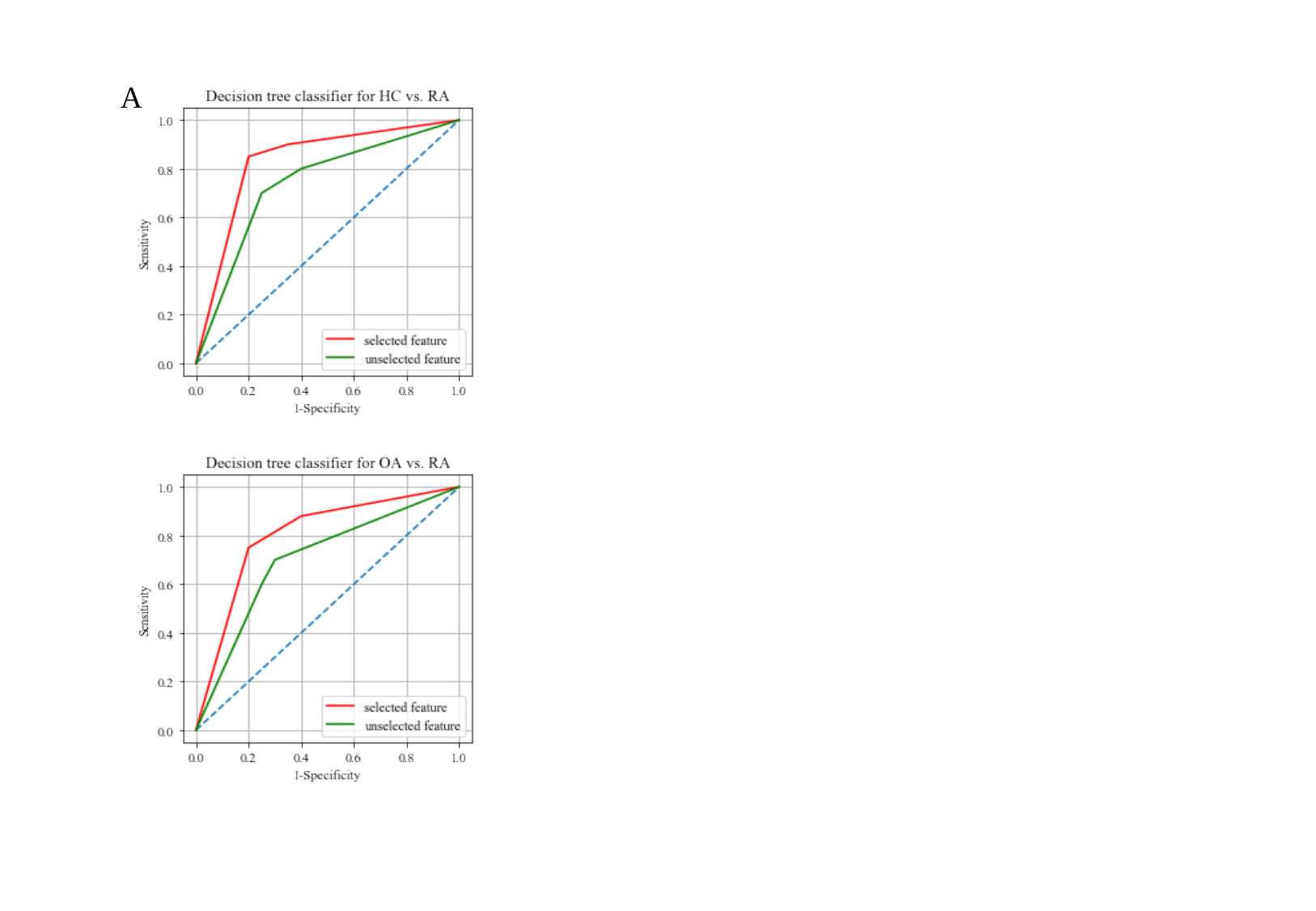

A

## Slide 2
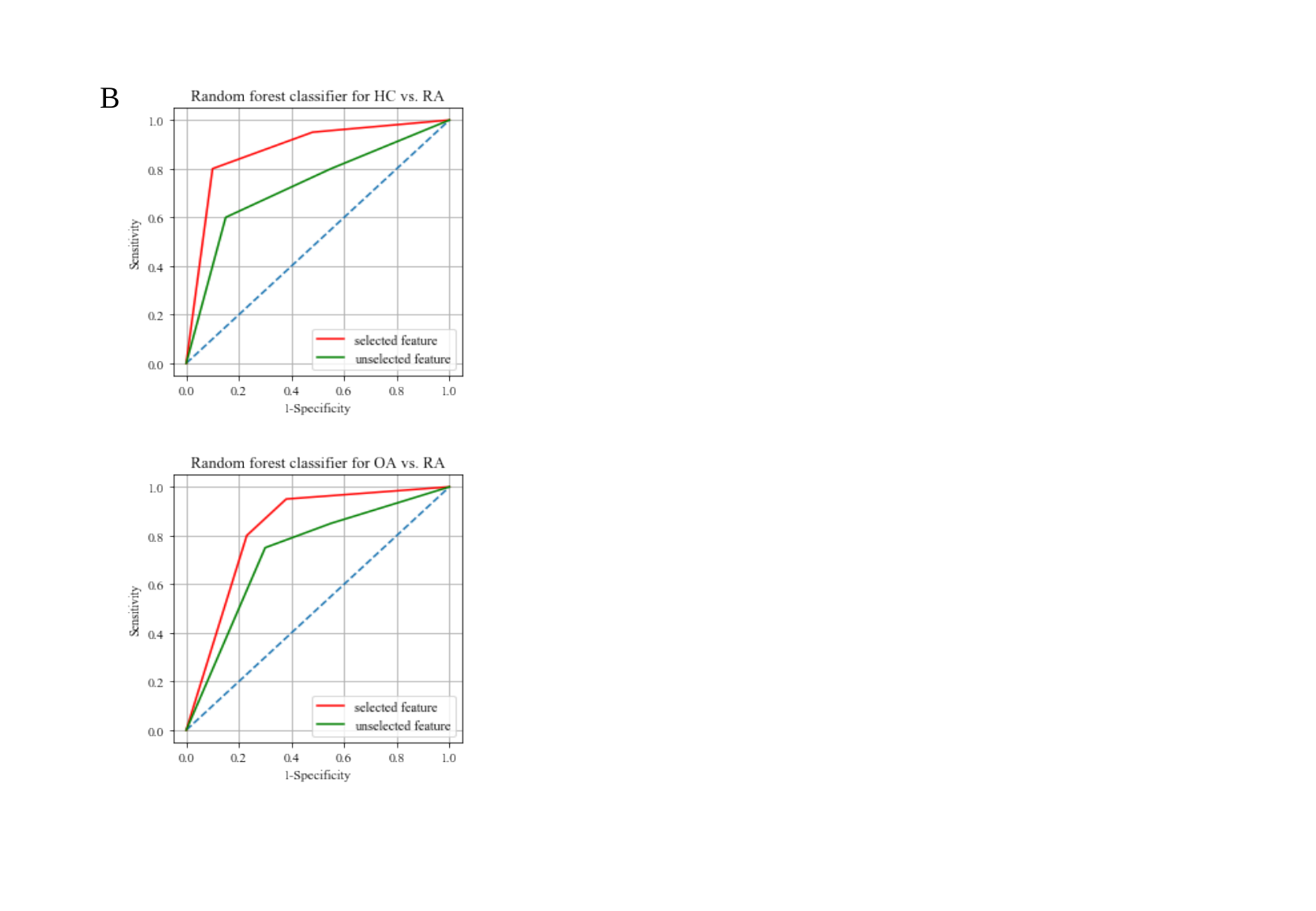

B

## Slide 3
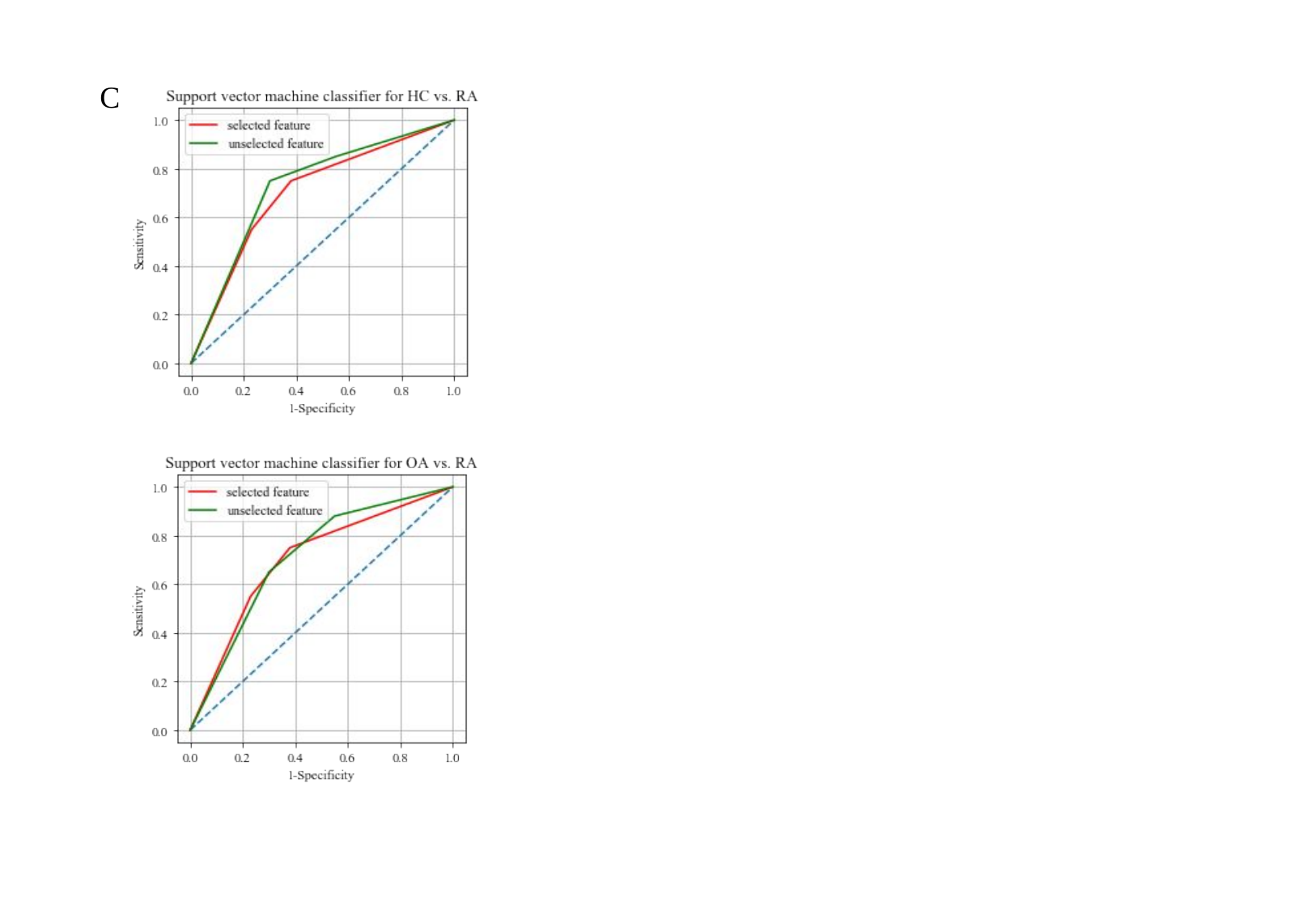

C

Supplement: Supplementary file 4 — Additional file 4: Fig. S2. Comparison of receiver operating characteristics (ROC) curves from unselected features and selected features in (A) decision tree, (B) random forest classifier, and (C) support vector machine classifier. [file 12911_2020_1380_MOESM4_ESM.pptx]
